# Sequential JAK inhibition enhances antitumor immunity after combined anti–PD-1 and anti-CTLA4

**DOI:** 10.1172/jci.insight.187921

**Published:** 2025-02-27

**Authors:** Marcel Arias-Badia, PeiXi Chen, Yee May Lwin, Aahir Srinath, Aram Lyu, Zenghua Fan, Serena S. Kwek, Diamond N. Luong, Ali Setayesh, Mason Sakamoto, Matthew Clark, Averey Lea, Rachel M. Wolters, Andrew Goodearl, Fiona A. Harding, Jacob V. Gorman, Wendy Ritacco, Lawrence Fong

**Affiliations:** 1 Division of Hematology/Oncology, Department of Medicine, University of California, San Francisco, San Francisco, California, USA.; 2 Immunotherapy Integrated Research Center, Fred Hutchinson Cancer Center, Seattle, Washington, USA.; 3 AbbVie Bioresearch Center, Worcester, Massachusetts, USA.; 4 AbbVie Redwood City, Redwood City, California, USA.; 5 Research and Development, AbbVie, North Chicago, Illinois, USA.; 6 Helen Diller Family Comprehensive Cancer Center, University of California, San Francisco, San Francisco, California, USA.

**Keywords:** Immunology, Oncology, Cancer immunotherapy, Cellular immune response, Drug therapy

## Abstract

While immune checkpoint inhibition (CPI) has reshaped cancer treatment, the majority of patients with cancer do not benefit from this approach, which can also cause immune-related adverse events. Induction of IFN-γ responses is thought be necessary for antitumor immunity, but growing evidence also implicates IFN-γ as a tumor-intrinsic mediator of CPI resistance. CPI-induced IFN-γ mediates activation-induced cell death in T cells as an immune-intrinsic mechanism of resistance. In this study, we found that transient block of IFN-γ signaling through administration of the JAK1 inhibitor ABT-317 enhanced antitumor T cell responses with CPI in preclinical models. Importantly, sequential but not concomitant ABT-317 treatment led to significantly reduced toxicity and improved tumor efficacy. Sequential treatment reduced activation-induced T cell death and enhanced expansion of tumor-reactive T cell subsets with increased effector function in vivo and ex vivo. Only CPI in combination with ABT-317 also enhanced memory responses by protecting mice from tumor rechallenge. These results demonstrate that JAK inhibition within a discrete time window following CPI addresses an immune-intrinsic mechanism of therapeutic resistance.

## Abstract 2

In this study, we describe how immune checkpoint inhibitors, drugs that fight cancer by reinvigorating the host immune system, can achieve improved responses in mice by preventing overwhelming T cell activation.

## Graphical abstract

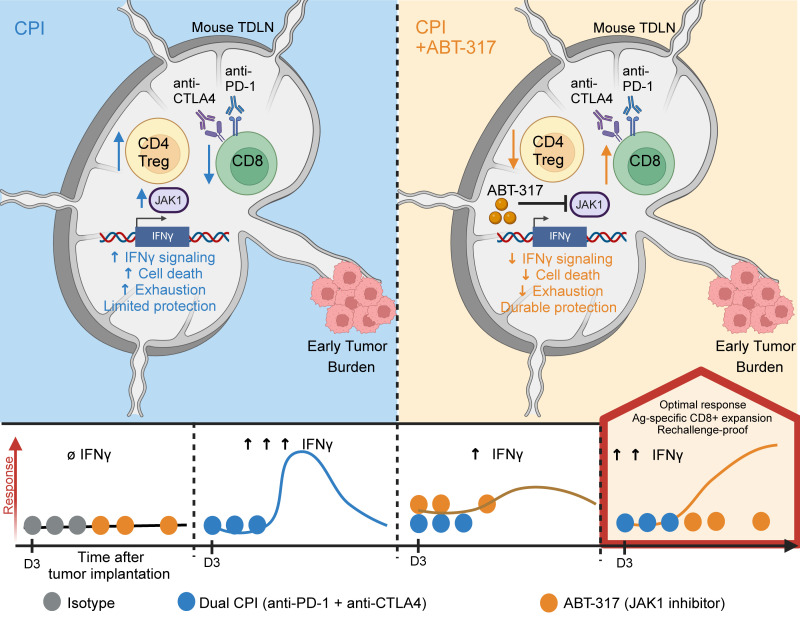

## Introduction

Immune checkpoint inhibition (CPI) represents a major cancer therapy. However, a substantial number of patients end up not responding at all or developing resistance to treatment. IFN-γ is a central cytokine in the immune system that provides a necessary signal for effective T cell antitumor responses (1). IFN-γ has also recently been identified as a key component for chimeric antigen receptor (CAR) T cell efficacy in solid tumors (2). On the other hand, overwhelming IFN-γ signaling in the tumor microenvironment can lead to defective antitumor responses because of loss of critical T cell clones and, eventually, resistance to immunotherapy (3–7). IFN-γ receptor signaling converges on the Janus kinase (JAK) family, which promotes the phosphorylation, dimerization, and nuclear translocation of members of the STAT transcription factor family, such as STAT1, STAT3, or STAT5, and the subsequent engagement of inflammatory programs (8, 9). Inhibitors of this pathway have been used to dampen pathologic inflammation in the context of transplantation or autoimmune disease (10–12). Emerging evidence in cancer indicates that tumors may be exploiting IFN-γ signaling to acquire immunosuppressive features that paradoxically resist antitumor immunity (13–15). Hence, several JAK inhibitors with varying selectivity have been developed or repurposed in the past for the treatment of cancer, mostly alone or in combination with chemotherapy (16). Ruxolitinib, a JAK1/2 inhibitor that was initially approved for the treatment of myelofibrosis and graft-versus-host disease, has been administered to patients with breast cancer in combination with anti–programmed cell death ligand 1 (anti–PD-L1) in an ongoing phase I clinical trial (NCT03012230), which set a promising precedent for the combination of JAK inhibitors and CPI. Recently published clinical trial data in refractory Hodgkin lymphoma (NCT03681561), first-line non–small cell lung cancer (NCT03425006), and CAR-treated acute myeloid leukemia patients (NCT03766126) have underscored the ability of JAK inhibition to improve T cell fitness and counter immunosuppressive tumor microenvironments when combined with immunotherapy (17–19). On the other hand, ruxolitinib has been associated with marked worsening of CD8^+^ T cell proliferation and survival (20). To better understand the interaction between JAK inhibition and CPI, we characterized early tumor burden (ETB) immune responses upon treatment with ABT-317, a JAK1 inhibitor (21, 22), and combined anti–PD-1 and anti–cytotoxic T lymphocyte antigen 4 (anti-CTLA4) therapy (jointly termed CPI onward) in the murine prostate TRAMP-C2 tumor model, which has previously shown low levels of response to CPI (3). We found that combining CPI with sequential, but not concomitant, administration of ABT-317 significantly impaired TRAMP-C2 tumor growth. Using ex vivo restimulation assays and time course multiomic characterization of antitumor T cell responses in mice, we found that ABT-317 prevented the early accumulation of CD4^+^ Tregs and protected tumor antigen CD8^+^ stimulator of prostatic adenocarcinoma-specific T cells-1–specific (SPAS-1–specific) T cells from IFN-γ–driven dysfunctional exhaustion and cell death in tumor-draining lymph nodes (TDLNs), leading to improved T cell fitness and even immunological memory upon TRAMP-C2 rechallenge. We cross-validated these findings by immunophenotyping colorectal MC38 tumors treated with CPI and ABT-317. In these experiments, we found a consistent, but more moderate, improvement of antitumor T cell fitness upon treatment with ABT-317, including the accumulation of progenitor exhausted T (Tpex) cells in the tumor microenvironment or the higher frequency and cytotoxic profile of MC38-reactive CD8^+^ ADP-dependent glucokinase–positive (ADPGK^+^) T cells, thereby demonstrating the importance of the temporal component for optimal immune responses upon CPI treatment followed by JAK inhibition. Overall, these findings reinforce the counterintuitive synergy that “immunosuppressive” JAK inhibition can have when combined with CPI at the right therapeutic time window and contribute to the optimization of tailored immunotherapies for prostate cancer and other malignancies.

## Results

### JAK inhibition synergizes with dual CPI only in sequential regime.

**Figure 1:**
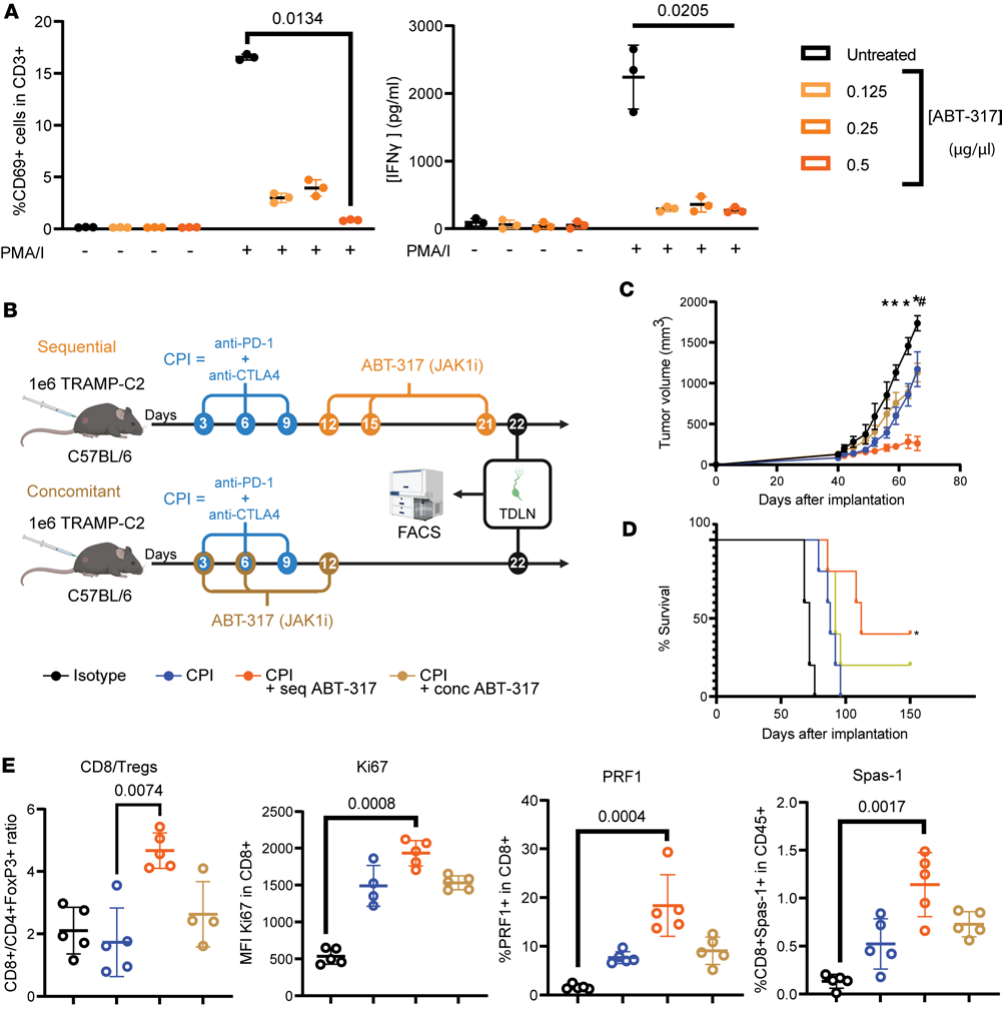
Sequential, but not concomitant, ABT-317 synergizes with CPI for improved antitumor efficacy. (**A**) A total of 10^5^ naive mouse splenocytes were stimulated with PMA-ionomycin for 4 hours in the presence of 0, 0.125, 0.25, or 0.5 μg/μL ABT-317. Plots show CD69^+^ T cells by flow cytometry and IFN-γ concentration in culture supernatants by ELISA. Significant *P* values by Kruskal-Wallis are shown. (**B**) Experiment design for sequential versus concomitant ABT-317 regimes in an early tumor burden (ETB) scenario. C57BL/6J mice implanted with 1 million (1M) mouse prostate adenocarcinoma TRAMP-C2 cells were treated with CPI (anti–PD-1 and anti-CTLA4) on days 3, 6, and 9 after implantation. In the sequential (seq) regime, mice were treated with ABT-317 on days 12, 15, and 21. In the concomitant (conc), mice were treated with ABT-317 on days 3, 6, and 12. While most mice were assessed for tumor growth, TDLNs from *n* = 3 animals from *n* = 2 experiments were harvested and immunophenotyped by flow cytometry. (**C**) Tumor volume curves for mice involved in **B** until day 66 after implantation. *, *P* < 0.05 CPI + ABT-317 (seq) vs. isotype by 1-way ANOVA applying Kruskal-Wallis; ^#^, *P* < 0.05 CPI + ABT-317 (seq) vs. all other groups by 1-way ANOVA applying Kruskal-Wallis. Error bars represent SEM. (**D**) Kaplan-Meier survival curves for all mice involved in **B**. *, *P* < 0.05 CPI + ABT-317 (seq) vs. CPI + ABT-317 (conc) by log-rank Mantel-Cox test. (**E**) Scatterplots showing flow cytometry parameters from TDLNs harvested on day 22 from the studies described in **B**. From left to right, CD8^+^/CD4^+^FoxP3^+^ (Treg) ratio, Ki67 MFI in gated CD8^+^ T cells, percentage of PRF1^+^ effector cells within CD8^+^ T cells, and percentage of TRAMP-C2 antigen-specific CD8^+^SPAS-1^+^ T cells within the CD45^+^ compartment. Significant *P* values obtained by Kruskal-Wallis are shown.

We have previously shown that dual CPI treatment with anti–PD-1 and anti-CTLA4 compromises antitumor immunity in the murine prostate cancer model TRAMP-C2 by inducing activation-induced cell death (AICD) in an IFN-γ–dependent fashion (3). We interrogated the ability of the JAK1 inhibitor ABT-317 to modulate the immune responses driven by dual CPI anti-CTLA4 and anti–PD-1 therapy. When added to in vitro PMA/ionomycin-stimulated naive C57BL/6J mouse splenocytes, ABT-317 was able to substantially decrease CD69 upregulation and IFN-γ release at a concentration of 0.125 μg/μL (Figure 1A). To study the immune-intrinsic component of ABT-317, we compared sequentially starting JAK inhibition 3 days after the last CPI dose versus concomitantly starting JAK inhibition simultaneously to CPI ABT-317 dosing schedules in the ETB setting of prostate TRAMP-C2 tumors in mice (Figure 1B). While sequential ABT-317 after CPI significantly controlled tumor growth and increased survival, concomitant ABT-317 and CPI did not provide any advantage in controlling tumor growth compared to CPI, with a nonsignificant survival improvement (Figure 1, C and D). We immunophenotyped TDLNs from these mice on day 22 after implantation. While relative CD8^+^ T cell counts were highest in CPI, followed by sequential CPI + ABT-317, we observed a reduction in TDLN overall CD4^+^ T cells and Tregs in sequential ABT-317 (Supplemental Figure 1A; supplemental material available online with this article; https://doi.org/10.1172/jci.insight.187921DS1), leading to significantly increased CD8/CD4 ratios in the sequential ABT-317 regime, together with higher levels of proliferating Ki67^+^, effector perforin 1–positive (PRF1^+^), and tumor-reactive CD39^+^ CD8^+^ T cells, as well as higher TRAMP-C2 antigen-specific CD8^+^SPAS-1^+^ T cells (Figure 1E and Supplemental Figure 1B). SPAS-1^+^ T cells from CPI + ABT-317–treated mice also showed significantly higher levels of Ki67 and PRF1, while SPAS-1^+^ T cells from CPI-treated mice showed significantly higher levels of PD-1 and a moderate increase in LAG-3 (Supplemental Figure 1C), both associated with T cell dysfunction and recently found to synergize in the maintenance of T cell exhaustion programs (23). These data highlight the time-sensitive, immune-intrinsic implications of disrupting the JAK signaling pathway in cancer immunotherapy.

### JAK1 inhibition ablates early IFN-γ–driven antitumor responses without compromising efficacy of CPI.

**Figure 2:**
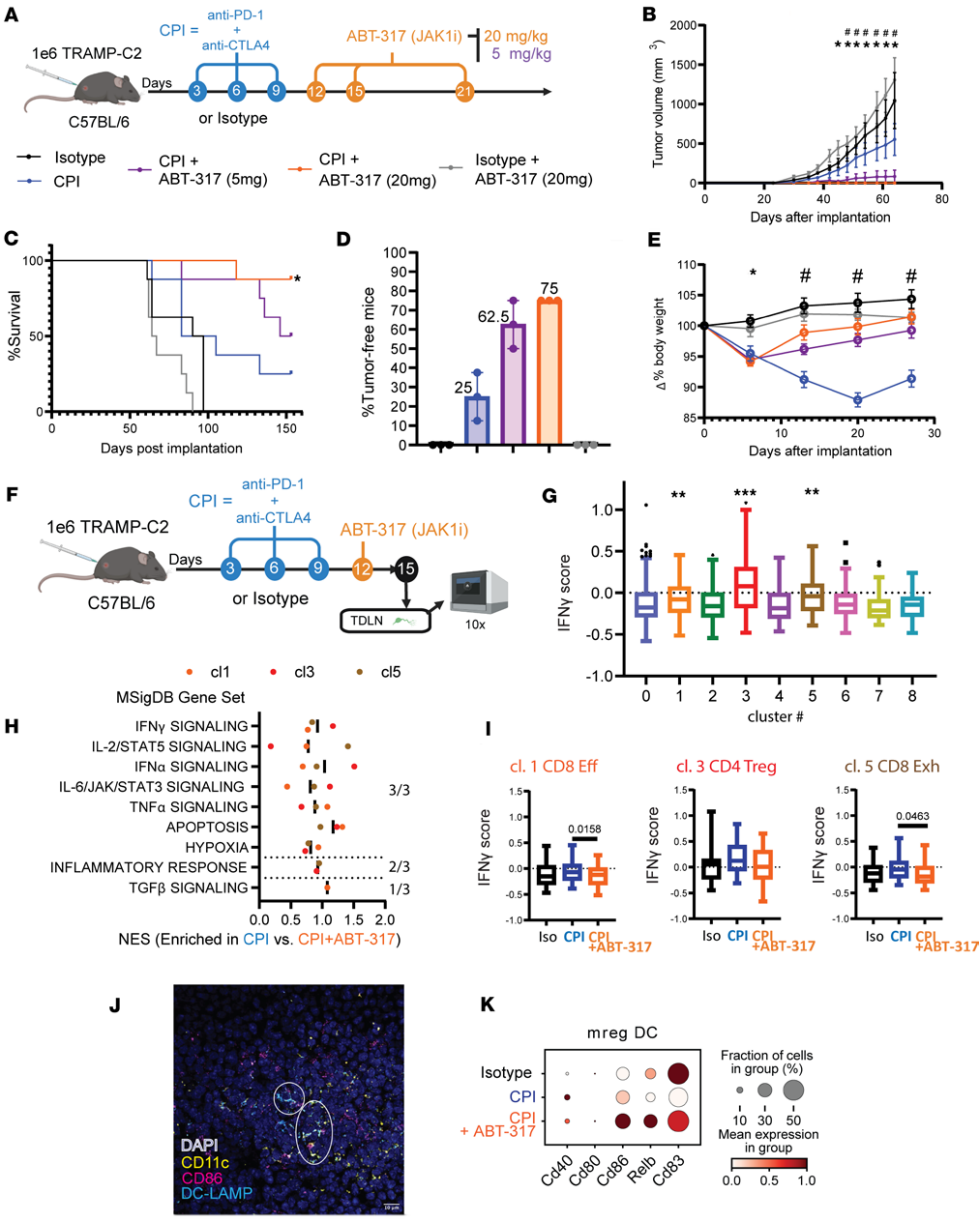
JAK inhibitor ABT-317 synergizes with CPI to achieve long-lasting antitumor efficacy in a dose-dependent fashion. (**A**) Treatment with CPI and ABT-317 in the ETB TRAMP-C2 model. TRAMP-C2 cells (1M) were implanted in the right flank of C57BL/6J mice. Antibodies were injected i.p. on days 3, 6, and 9. ABT-317 (20 or 5 mg/kg) was injected i.p. on days 12, 15, and 21. (**B**) Tumor volume curves for *n* = 1 of 3 experiments. *, *P* < 0.05 isotype vs. CPI + ABT-317 (20 and 5 mg) by Kruskal-Wallis; ^#^, *P* < CPI vs. CPI + ABT-317 (20 and 5 mg) by Kruskal-Wallis. Error bars represent SEM. (**C**) Kaplan-Meier survival curves. *, *P* < 0.05 CPI + ABT-317 vs. all by Mantel-Cox. (**D**) Percentage of tumor-free mice (*n* = 3 experiments). (**E**) Percentage body weight variation. *, *P* < 0.05 isotype and isotype + ABT-317 vs. CPI and CPI + ABT-317 (20 and 5 mg) by Kruskal-Wallis; ^#^, *P* < CPI vs. CPI + ABT-317 (20 mg) by Kruskal-Wallis. (**F**) TDLN immunophenotyping strategy (*n* = 3 mice per group, *n* = 2 experiments) by scRNA-Seq. (**G**) IFN-γ scores in TDLN T cells split by Leiden phenotypic clusters 0–8. Boxes cover interquartile interval, split by the median. Whiskers represent min to max values. Tukey outliers are shown. **, *P* < 0.05 vs. 0, 2, 4, 6, 7 by Tukey; ***, *P* < 0.0001 vs. all. (**H**) Enriched pathways in CPI vs. CPI + ABT-317 TDLN T cells from clusters 1, 3, and 5 by preranked GSEA from upregulated genes by differentially expressed genes (DEGs) precut by *P* < 0.05. NES, normalized enrichment score. (**I**) IFN-γ scores in scRNA-Seq TDLN T cell clusters 1, 3, and 5, split by treatment. Significant *P* values by Tukey are shown. Boxes cover interquartile interval, split by the median. Whiskers represent min to max values. (**J**) Representative immunofluorescence staining of TDLNs showing the presence of CD11c^+^CD86^+^DC-LAMP^+^ mreg DCs. (**K**) Dot plot matrix showing log-transformed gene expression of *Cd40*, *Cd80*, *Cd86*, *Relb*, and *Cd83* in TDLN mreg DCs.

When administered to mice challenged with TRAMP-C2 tumors receiving dual CPI, ABT-317 showed a significant dose-dependent improvement in tumor control and survival compared with dual CPI alone (Figure 2, A–C), rendering up to 75% of mice free of tumors at the higher dose of 20 mg/kg ABT-317 (Figure 2D). ABT-317 was also able to neutralize body weight loss observed in mice treated with CPI alone (Figure 2E). Notably, ABT-317 alone did not have any impact on tumor growth. To characterize early immune events leading to the observed tumor protection, we immunophenotyped TDLNs from mice on day 15 after implantation by single-cell RNA sequencing (scRNA-Seq) (Figure 2F). Though it was populated mainly by B cells (Supplemental Figure 2, A–C), we isolated the T cell compartment and identified 9 phenotypic clusters, including naive, regulatory, exhausted, and effector CD4^+^ and/or CD8^+^ T cell populations (Supplemental Figure 2, D–F). To identify the main downstream effects of JAK1 inhibition by ABT-317, we scored T cells for their normalized expression of a set of 26 previously published genes associated with IFN-γ signaling (24), including *Isg15*, *Ifngr1*, *Ccl5*, *Icos*, or *Stat1*, among others, and found a significantly increased “IFN-γ score” associated with treatment with CPI that was neutralized by the addition of ABT-317 at a pan-T cell level (Supplemental Figure 2, G and H). We found clusters 1, 3, and 5, composed by CD8^+^ effector T cells, CD4^+^ Tregs, and CD8^+^ exhausted T cells, respectively, to have significantly increased IFN-γ scores (Figure 2G). We performed differential gene expression (DGE) followed by gene set enrichment analysis (GSEA) between CPI and CPI + ABT-317 T cells from these clusters and found up to 7 enriched inflammatory pathways in all 3 clusters: IFN-γ, STAT5, IFN-α, JAK/STAT3, TNF-α, apoptosis, and hypoxia (Figure 2H). We consequently found significantly enriched IFN-γ scores in effector and exhausted CD8^+^ T cells (cl. 1 and 5) from CPI-treated mice and a nonsignificant trend (*P* = 0.0643) in CD4^+^ Tregs (cl. 3) (Figure 2I). Further analysis of the myeloid compartment revealed several DC subpopulations, including conventional DC1s (cDC1s), cDC2s, mature DCs enriched in immunoregulatory molecules (mreg DCs), and plasmacytoid DCs (Supplemental Figure 2, I–L). Given the role of mreg DCs in enhancing T cell antitumor activity in the tumor microenvironment (25, 26), we hypothesized that JAK1 inhibition might influence gene expression in these cells. We verified the presence of mreg DCs in TDLNs by immunofluorescence (Figure 2J), which showed decreased expression of DC maturation genes *Cd80* and *Cd86* in mice treated with CPI without ABT-317 (Figure 2K), suggesting impaired antigen presentation occurring in TDLNs from mice treated with CPI alone. These data demonstrate the multicellular effects of JAK inhibition in early antitumor responses.

### JAK1 inhibition after CPI leads to delayed antigen-specific T cell expansion with improved fitness.

**Figure 3:**
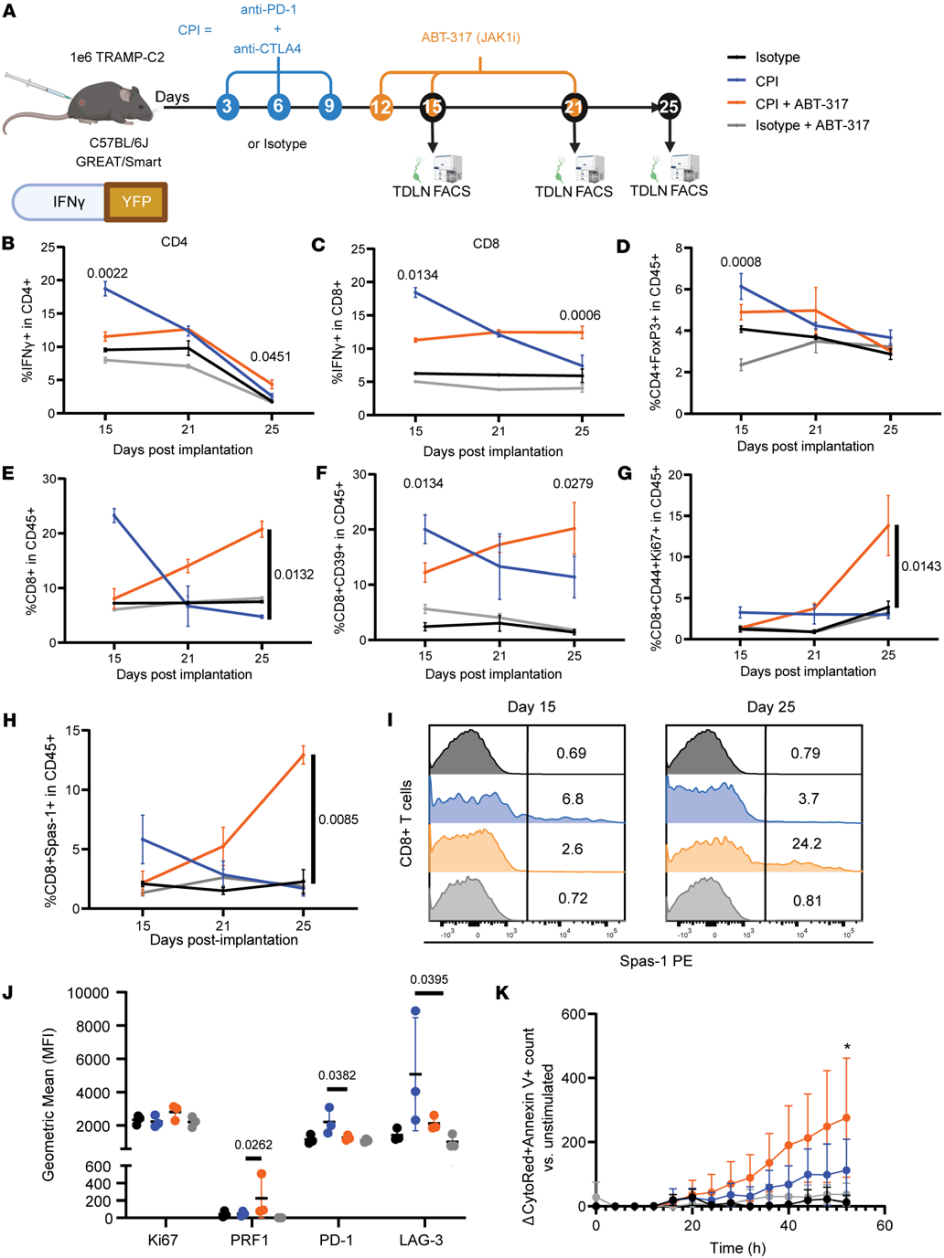
ABT-317 attenuates early IFN-γ–driven responses elicited by CPI to provide delayed expansion of tumor-reactive T cells. (**A**) Time course immunophenotyping of T cell responses in the ETB TRAMP-C2 model. IFN-γ–YFP reporter GREAT/Smart mice implanted s.c. with 1M TRAMP-C2 cells were treated i.p. with CPI alone or in combination with ABT-317 (20 mg/kg). TDLNs (*n* = 3 per group) were harvested on days 12, 15, 21, and 25. (**B**) Percentage of YFP^+^ (IFN-γ^+^) CD4^+^ T cells. Significant *P* values between groups in each time point by Kruskal-Wallis. (**C**) Percentage of YFP^+^CD8^+^ T cells. Significant *P* values between groups in each time point by Kruskal-Wallis. (**D**) Percentage of CD4^+^FoxP3^+^ Tregs within CD45^+^ cells. Significant *P* values between groups in each time point by Kruskal-Wallis. (**E**) Percentage of overall CD8^+^ T cells within the CD45^+^ compartment. Significant *P* values between groups in each time point by Kruskal-Wallis. (**F**) Percentage of tumor-reactive CD8^+^CD39^+^ T cells within CD45^+^ cells. Significant *P* values between groups in each time point by Kruskal-Wallis. (**G**) Percentage of proliferating and activated CD8^+^CD44^+^Ki67^+^ T cells within the CD45^+^ compartment. Significant *P* values between groups in each time point by Kruskal-Wallis. (**H**) Percentage of TRAMP-C2 antigen-specific CD8^+^SPAS-1^+^ T cells within the CD45^+^ compartment. Significant *P* values between groups in each time point by Kruskal-Wallis. (**I**) Representative mode-normalized histograms showing MFI of SPAS-1^+^ staining in gated CD8^+^ T cells from TDLNs on days 15 and 25. Values represent percentages of SPAS-1^+^ cells. (**J**) Geometric mean fluorescence intensity (MFI) of Ki67, PRF1, PD-1, and LAG3 in CD8^+^SPAS-1^+^ T cells from day 25 TDLNs. Significant *P* values by Kruskal-Wallis. (**K**) TRAMP-C2 cell death by total counts versus time (hours) (*n* = 2 experiments) (see Methods), normalized vs. unstimulated controls. *, *P* < 0.05 CPI + ABT-317 (orange) vs. isotype (black) by Kruskal-Wallis.

Because improved tumor control in the context of IFN-γ signaling inhibition is relatively counterintuitive, we aimed to dissect the underlying T cell dynamics behind the therapeutic efficacy of sequential ABT-317 after CPI. Thus, we immunophenotyped the T cell compartment in TDLNs from IFN-γ–reporter (yellow fluorescent protein, YFP) GREAT/Smart mice treated with dual CPI alone or in combination with ABT-317 on days 15, 21, and 25 after tumor implantation (Figure 3A). Overall CD4^+^ and CD8^+^ IFN-γ^+^ frequencies showed a declining trend in CPI-treated animals after an early significant increase, reaching down to isotype levels by day 25 (Figure 3, B and C). Notably, while CPI + ABT-317 treatment showed moderate early increases in both CD4^+^ and CD8^+^ IFN-γ^+^ T cell frequencies compared with isotype-treated animals, there was no apparent drop in CD8^+^IFN-γ^+^ T cells by day 25. We observed a similar declining pattern for CD4^+^ Tregs in CPI-treated mice, with more moderate early increases in the presence of ABT-317 (Figure 3D). CPI-treated TDLNs also showed a drastic early increase in CD8^+^ T cell frequencies, apparently blunted by the addition of ABT-317, which led to inverse frequencies by day 25, showing significantly CD8^+^-enriched TDLNs in CPI + ABT-317–treated animals (Figure 3E). We observed a very similar pattern when looking at tumor-reactive CD39^+^ or proliferating activated CD44^+^Ki67^+^CD8^+^ T cells, suggesting a shift in the timing of CPI-induced T cell expansion dependent of ABT-317 (Figure 3, F and G). We interrogated TDLN-derived T cells obtained by scRNA-Seq with a T cell exhaustion (Texh) score comprising known immune checkpoint genes like *Pdcd1*, *Tigit*, *Lag3*, *Tox*, among others (Supplemental Figure 3B). The resulting Texh score, led by elevated expression of *Pdcd1* and *Tox*, a reported orchestrator of terminal T cell exhaustion (27, 28), was significantly higher in CPI treatment alone, while addition of ABT-317 notably kept exhaustion in TDLN T cells at the isotype level (Supplemental Figure 3, B and C). According to our scRNA-Seq data, this CPI-specific effect seemed to be particularly affecting CD4^+^ Tregs by day 15, with subtle differences in CD8^+^ T cells (Supplemental Figure 3B), though we did not detect consequences on the effector T cell gene expression between groups (Supplemental Figure 3D). To better capture tumor antigen-driven T cell responses, we stained TDLN T cells reactive to the MHC class I TRAMP-C2 immunodominant antigen SPAS-1 (29). We found a very similar time course trend in the frequency of TDLN SPAS-1–reactive CD8^+^ T cells to overall CD8^+^ T cells and CD4^+^ Tregs, with an early CPI-induced boost, detectable prior to the treatment with ABT-317 by day 12 (Supplemental Figure 3E) that declined with time, and a delayed boost in CPI + ABT-317–treated animals. Strikingly, SPAS-1–reactive T cells accounted for up to 24.2% of CD8^+^ T cells and up to 12.5% of all immune cells in TDLNs in these mice by day 25 (Figure 3, H and I), demonstrating a delayed antigen-driven T cell expansion. Consequently, CPI-ABT-317 SPAS-1^+^ T cells from day 25 showed significantly increased PRF1 and reduced exhaustion markers PD-1 and LAG-3 compared with CPI alone (Figure 3J). Immune infiltrates from endpoint TRAMP-C2 tumors involved in these experiments did not show significant reminiscences of the early events occurring in the TDLNs from these mice involving the boost in CD4^+^ Tregs, overall CD8^+^ T cells, or SPAS-1^+^ T cells (Supplemental Figure 3, F–H). To validate the tumor-killing fitness of TDLN-derived T cells upon treatment with CPI alone or with ABT-317, we set up ex vivo tumor rechallenge assays after pulsing day 25 TDLN T cells with the MHC class I TRAMP-C2 tumor antigen SPAS-1 and coculturing them with target TRAMP-C2 cells. Interestingly, T cells from mice treated with CPI in combination with ABT-317 were able to kill target cells at significantly higher rates after 2-day cultures than T cells from CPI alone (Figure 3K). These observations reinforce the notion that, while minimal IFN-γ signaling is mandatory for an effective response against tumors, preventing its overwhelming activation can provide improved efficacy and CPI-induced antitumor T cell fitness.

### JAK1 inhibition prevents CPI-induced early T cell death.

**Figure 4:**
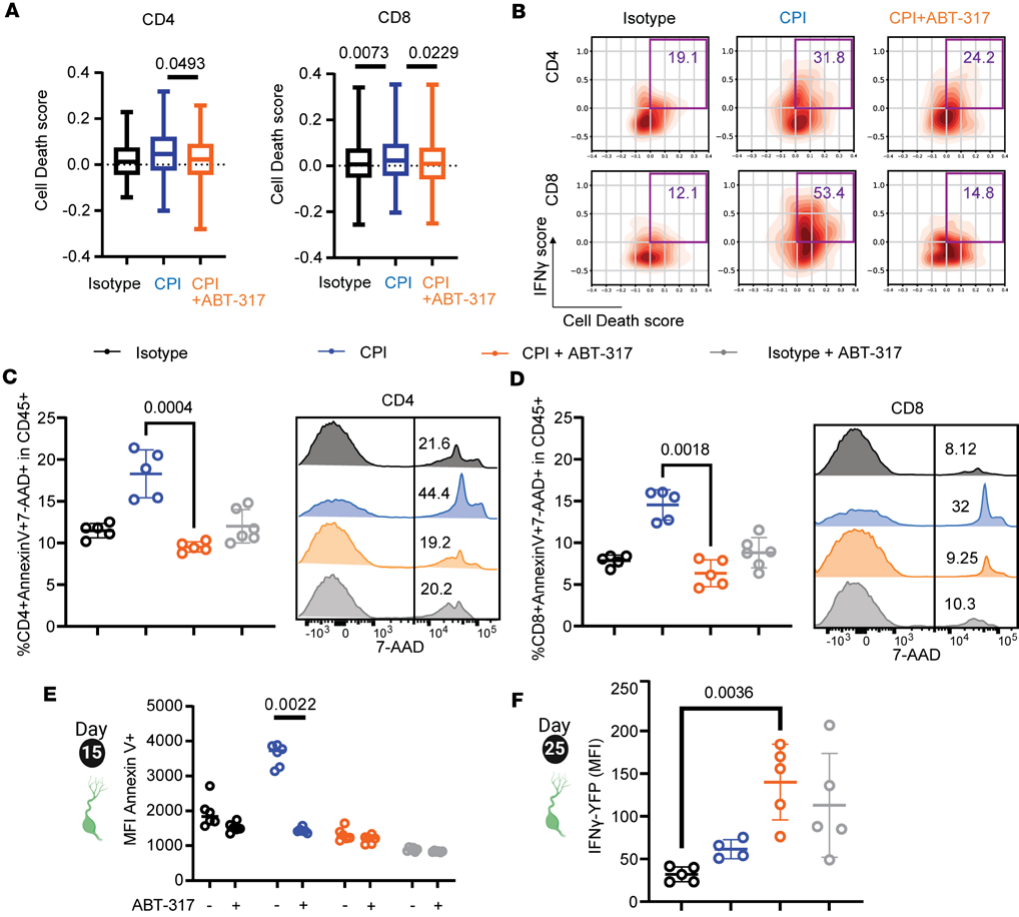
ABT-317 prevents early T cell death and leads to delayed, improved T cell fitness. (**A**) Cell death scores by scRNA-Seq in TDLN CD4^+^ and CD8^+^ T cells, split by treatment. Significant *P* values by Tukey are shown. Boxes cover interquartile interval, split by the median. Whiskers represent min to max values. (**B**) Representative density plots showing scRNA-Seq cell death scores (*x* axis) versus IFN-γ scores (*y* axis) in TDLN CD4^+^ and CD8^+^ T cells, by treatment. Gates (purple) contain all cells with scores > 0.0 for both gene sets. Values represent percentage. (**C**) Left, percentage of apoptotic annexin V^+^7-AAD^+^CD4^+^ T cells within the CD45^+^ compartment for TDLN T cells harvested on day 15 as pictured in Figure 3A. Significant *P* values by Kruskal-Wallis are shown; right, representative histograms showing 7-AAD MFI in gated CD4^+^ T cells. Values represent percentages. (**D**) Same as **C**, for CD8^+^ TDLN T cells. (**E**) Day 15 TDLN-derived T cells from the study shown in Figure 3A were stimulated with PMA-ionomycin in the presence or absence of ABT-317 in 96-well plates for 4 hours, after which wells were stained for annexin V and analyzed by flow cytometry. Scatterplot showing annexin V MFI. Significant *P* values by Kruskal-Wallis are shown. (**F**) Day 25 TDLN-derived T cells were stimulated with CD3/CD28 beads in 96-well plates for 48 hours and then analyzed by flow cytometry. Scatterplot showing YFP (IFN-γ) MFI within CD8^+^ T cells. Significant *P* values by Kruskal-Wallis are shown. Error bars represent SD.

To assess how much the drop in T cell frequencies observed in mice treated with CPI alone could be explained by previously reported clonal deletion (3), we interrogated day 15 TDLN T cells for cell death–associated gene expression, including members of the caspase family, with a 45-gene “cell death” set (Supplemental Figure 4, A and B). CD4^+^ Tregs (cluster 3) showed the highest death score, followed by effector T cells (cluster 1) (Supplemental Figure 4C). When compared by treatment, both CPI-treated CD4^+^ and CD8^+^ T cells showed a significantly higher cell death score, which correlated with higher IFN-γ score rates in TDLN T cells, with bigger differences in the CD8^+^ compartment (Figure 4, A and B, and Supplemental Figure 4D). We assessed apoptosis rates in T cells from day 15 TDLNs by flow cytometry staining of annexin V (early apoptotic) and 7-AAD (late apoptotic) (Supplemental Figure 4E). Concordant with our scRNA-Seq data, we found a significant increase in late apoptotic CD4^+^ T (up to 21.4% of all immune cells) and CD8^+^ T cells (up to 16.07% of all immune cells) in mice treated with CPI alone, while treatment with ABT-317 was able to keep T cell apoptosis rates — both CD4 and CD8 — at isotype control levels (Figure 4, C and D). We hypothesized that the increase in early cell death in CPI alone would render those T cell populations differentially sensitive to subsequent stimulations. To test this, we isolated equal numbers of viable T cells from day 15 TDLNs of mice, restimulated them ex vivo with PMA-ionomycin, and assessed annexin V expression by flow cytometry after 4 hours. Strikingly, only T cells from TDLNs of mice treated with CPI alone showed significantly increased apoptotic levels after indiscriminate PMA-ionomycin restimulation, which was in turn prevented by exogenous addition of ABT-317 to the cultures (Figure 4E). To test the long-term impact on T cell fitness, we tested the ability of day 25 TDLN-derived T cells to express IFN-γ after CD3/CD28 ex vivo restimulation. T cells from mice treated with CPI alone showed a significant defect in IFN-γ levels after CD3/CD28 ex vivo restimulation compared with T cells from mice treated with CPI + ABT-317 (Figure 4F). These findings underscore the detrimental effects derived from excessive T cell activation in this ETB model, and importantly, demonstrate the possibility to identify those events systemically by analyzing lymphoid organs when there is no accessible or practicable tumor material.

### JAK1 inhibition after CPI confers long-lasting immunological memory.

**Figure 5:**
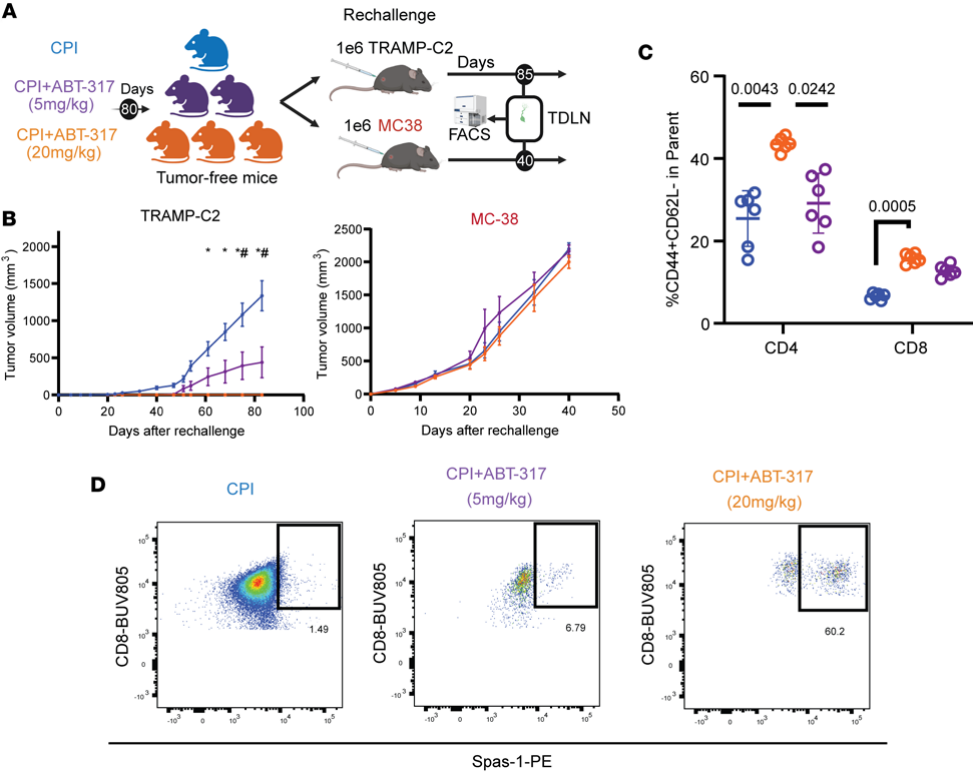
ABT-317 provides protective immunological memory against tumor rechallenge in mice treated with CPI. (**A**) Tumor rechallenge experiment design. Briefly, tumor-free animals from *n* = 2 studies (see Methods) described in Figure 2A were challenged with a second round of either 1M related TRAMP-C2 or 1M unrelated mouse colorectal MC38 tumor cells on their left flanks. TDLNs were harvested and analyzed by flow cytometry on day 40 (MC38) or day 85 (TRAMP-C2). (**B**) Tumor volume curves for implantations described in **A**: second challenge of TRAMP-C2 (left) or unrelated MC38 (right). *, *P* < 0.05 CPI vs. CPI + ABT-317 (20 mg) by 1-way ANOVA applying Kruskal-Wallis; ^#^, *P* < 0.05 CPI vs. CPI + ABT-317 (20 and 5 mg) by 1-way ANOVA applying Kruskal-Wallis. Error bars represent SEM. (**C**) Percentage of CD44^+^CD62L^–^ TDLN T cells by flow cytometry from TRAMP-C2 rechallenged mice involved in the study described in **A**, split into CD4^+^ and CD8^+^ compartments. Significant *P* values obtained by 1-way ANOVA applying Kruskal-Wallis are shown. Error bars represent SD. (**D**) Representative flow cytometry dot plots showing CD8^+^SPAS-1^+^ T cells from TDLNs of TRAMP-C2 rechallenged mice involved in **A**. Values represent percentages.

After the striking 75% tumor-free mice upon CPI + ABT-317 treatment (Figure 2D), we wondered if and how much JAK inhibition contributed to the reshaping of the immune system, so we assessed tumor outcomes and T cell responses against related (TRAMP-C2) or unrelated (MC38) tumor rechallenges in these mice without additional treatments (Figure 5A). Expectedly, no effect in tumor growth was observed in MC38-challenged mice. However, there was a dose-dependent ABT-317–related protection against TRAMP-C2 rechallenges, with mice previously treated with high-dose ABT-317 not developing TRAMP-C2 tumors at all compared with mice treated with CPI alone or in combination with low-dose ABT-317, suggesting long-lasting immunological memory by JAK inhibition (Figure 5B). We performed multiparameter flow cytometry on T cells from the TDLNs of the rechallenged mice and quantified CD44^hi^CD62L^–^ effector memory T cell (Tem) frequencies, which were clearly increased in TRAMP-C2–rechallenged TDLNs (Supplemental Figure 5, A and B). In these, we observed significantly higher Tem frequencies in rechallenged mice originally treated with CPI + high-dose ABT-317, in correlation with response (Figure 5C), though these differences could not be observed in day 15 TDLN scRNA-Seq (Supplemental Figure 5C), likely owing to delayed contraction of Tems in CPI-treated animals. We also observed a notable increase in TDLN CD8^+^SPAS-1^+^ T cell frequencies in CPI + high-dose ABT-317 rechallenged mice (Figure 5D). These antigen-specific cells also showed significantly higher levels of CD44 and trends for lower CD62L, as well as elevated proliferation Ki67, compared with the other 2 groups, suggesting reinforced memory against the TRAMP-C2 antigen SPAS-1 (Supplemental Figure 5D). These observations highlight the potential long-term impact of early IFN-γ modulation in the outcome of tumors and sustained surveillance after immune checkpoint inhibition.

### JAK1 inhibition negates the CPI-associated IFN-γ program in tumors.

**Figure 6:**
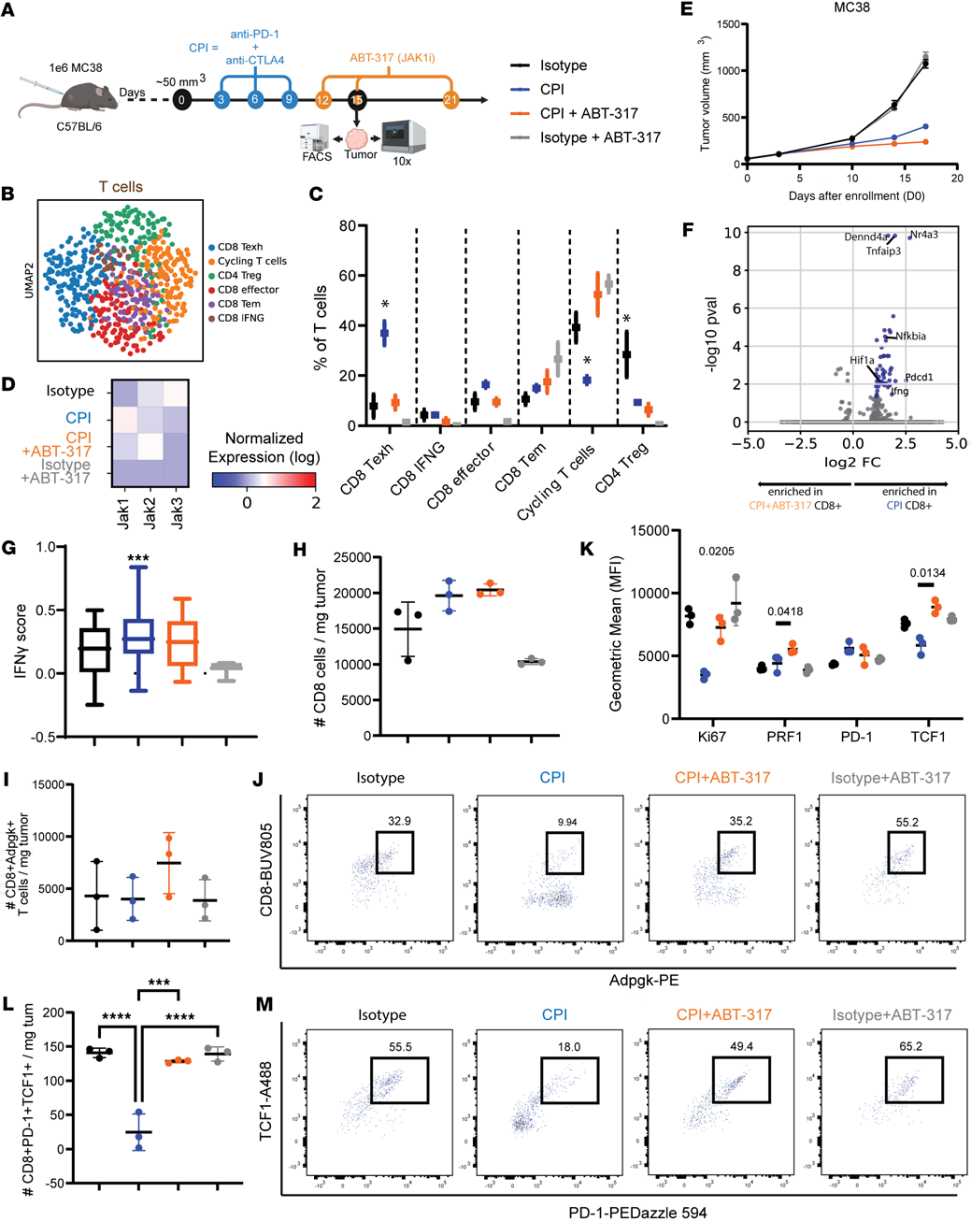
JAK inhibition attenuates IFN-γ–responsive signatures in the tumor microenvironment. (**A**) Study of CPI and ABT-317 in a minimal-burden tumor model. Briefly, 1M MC38 cells were implanted in the right flank of C57BL/6J mice. Mice were enrolled into treatment groups when tumors reached 50 mm^3^ (day 0). Antibodies were injected i.p. on days 3, 6, and 9. ABT-317 (20 mg/kg) was injected i.p. on days 12, 15, and 21. Tumors (*n* = 3 per group) were harvested on day 15 for scRNA-Seq, while *n* = 5 mice were assessed for tumor growth. (**B**) Tumor volume curves for mice involved in *n* = 1 of 2 experiments. Error bars represent SEM. (**C**) Uniform manifold approximation and projection showing 2,432 MC38 tumor-infiltrating T cells, distributed in 6 phenotypic clusters by Leiden (res. 0.5). (**D**) Heatmap showing log-transformed expression of *Jak1*, *Jak2*, and *Jak3* in MC38-infiltrating T cells. (**E**) T cell phenotypic cluster frequencies by treatment group. *, *P* < 0.05 vs. rest by Kruskal-Wallis. (**F**) Volcano plot showing DEGs in tumor-infiltrating CD8^+^ T cells from CPI-treated mice. Significantly enriched genes in blue. (**G**) IFN-γ score in tumor-infiltrating T cells. ***, *P* = 0.0409 by 1-way ANOVA. (**H**) CD8^+^ T cell counts/mg of tumor. (**I**) CD8^+^ADPGK^+^ T cell counts/mg of tumor. (**J**) Representative flow cytometry dot plots showing MC38 antigen-reactive CD8^+^ADPGK^+^ T cells from MC38 tumors. Values represent percentages. (**K**) MFI of Ki67, PRF1, PD-1, and TCF1 in tumor-infiltrating CD8^+^ADPGK^+^ T cells. Significant *P* values by Kruskal-Wallis are shown. (**L**) CD8^+^PD-1^+^TCF1^+^ (Tpex) cell counts/mg of tumor. ***, *P* < 0.001 by Kruskal-Wallis; ****, *P* < 0.0001 by Kruskal-Wallis. (**M**) Representative flow cytometry dot plots showing tumor-infiltrating Tpex cells. Values represent percentages.

To capture events taking place during CPI and ABT-317 treatments in the tumor microenvironment, we implanted MC38 tumors in mice and enrolled them into treatments after a minimal tumor burden of 50 mm^3^ was achieved (Supplemental Figure 6A). We then evaluated tumor outcomes and immunophenotyped MC38 tumors obtained on day 15 after implantation by flow cytometry and scRNA-Seq (*n* = 3 per group) (Figure 6A). In this setting, CPI + ABT-317 provided nonsignificant higher antitumor efficacy and overall survival to CPI alone (Figure 6B and Supplemental Figure 6A). Our scRNA-Seq analysis on tumors identified 6 major cell populations, including MC38, fibroblast, endothelial, cycling CD45^–^, myeloid, and T cells (Supplemental Figure 6B). T cell unsupervised subclustering resulted in 6 phenotypic T cell clusters comprising *Cd4*^+^ Tregs, *Cd8a*^+^*Ifng*^hi^ exhausted and nonexhausted T cells, and effector T and Tem cells, as well as a mixed cluster of cycling T cells (Figure 6C and Supplemental Figure 6, C and D). ABT-317 target *Jak1* showed increased expression in T cells upon CPI treatment, though addition of ABT-317 brought it to isotype levels. *Jak2* upregulation observed in CPI-ABT-317–treated mice might suggest ongoing compensation mechanisms (Figure 6D). We observed significantly enriched proportions of exhausted CD8^+^ T cells and decreased cycling T cells in CPI-treated tumors by scRNA-Seq, in line with previous characterization of T cell dynamics in TDLNs (Figure 6E). Moreover, DGE between CPI and CPI + ABT-317 CD8^+^ T cells revealed upregulation of *Ifng* itself plus several IFN-γ–associated genes reportedly implicated in antitumor CD8^+^ and CAR T cell dysfunction like *Pdcd1*, *Dennd4a* (30), *Tnfaip3* (31, 32), and *Nr4a3* (33, 34) or the hypoxia-related transcription factor *Hif1a*, also associated with T cell exhaustion (35) and in line with the enriched pathways found in TDLNs (Figure 6F). Accordingly, IFN-γ score was significantly higher in T cells from CPI-treated tumors (Figure 6G), particularly both in CD8^+^ exhausted (cluster 0) and CD8^+^ effector (cluster 3) T cells, followed by CD4^+^ Tregs (Supplemental Figure 6E). Interestingly, we also found elevated cell death scores in CD8^+^ cluster 0, though no significant differences were reached when compared by treatment (Supplemental Figure 6F). Parallel flow cytometry analysis revealed significantly increased CD4^+^ Tregs in CPI-treated tumors (Supplemental Figure 6G), with isotype showing the highest frequency by scRNA-Seq, followed again by CPI. Although overall CD8^+^ T cell tissue-specific counts were highest and did not differ between CPI- and CPI + ABT-317–treated groups by flow cytometry (Figure 6H), we captured antigen-specific T cell responses by flow cytometry staining with a tetramer against the MC38-specific MHC class I antigen ADPGK (36), and we found increased tissue-specific CD8^+^ADPGK^+^ counts in tumors from CPI + ABT-317–treated mice (Figure 6, I and J). Notably, ADPGK^+^ T cells from these mice showed significantly greater proliferation (Ki67), cytotoxicity (PRF1), and self-renewal potential (TCF1) than tumor-infiltrating ADPGK^+^ T cells from CPI-treated animals, suggesting sustained antigen-specific T cell expansion favored by ABT-317 (Figure 6K). In fact, overall CD8^+^PD-1^+^TCF1^+^ Tpex cells were depleted in CPI-treated tumors (Figure 6, L and M), thereby potentially affecting additional T cell populations. Our tumor-infiltrating myeloid scRNA-Seq analysis revealed improved DC maturation and antigen presentation potential in tumors treated with CPI + ABT-317 in the form of upregulated *Cd80*, *Cd83*, and *Cd86*, in line with our observations in the TDLNs of TRAMP-C2–challenged mice (Supplemental Figure 6, H–L). Overall, these findings underscore the time-sensitive benefits of IFN-γ modulation in boosting antigen-driven T cell responses against tumors.

## Discussion

IFN signaling is an essential feature of successful antitumor immune responses (1, 37), with particular relevance in immunotherapies targeting solid tumors (2). However, when overwhelmingly unleashed, it is also a source of AICD (5) and T cell dysfunction (7, 38). We have previously demonstrated that excessive IFN-γ in ETB settings leads to clonal T cell loss and subsequent resistance to CPI in the prostate cancer model TRAMP-C2 (3). In this study, because IFN-γ signaling converges into the JAK/STAT pathway (39), we evaluated the ability of the JAK1 inhibitor ABT-317 to modulate early IFN-γ downstream effects upon tumor challenge and its implications in preclinical prostate tumor outcomes and resulting immune status when combined with dual anti-CTLA4 + anti–PD-1 treatment. This constitutes a likely unprecedented use for this compound in prostate cancer. In our ETB model, we found ABT-317 to provide dose-dependent, toxicity-free advantage in controlling TRAMP-C2 tumors only when administered sequentially after dual CPI, rendering most of challenged mice tumor free and better immunologically prepared to reject secondary, antigen-related tumor challenges. We consider the temporal component of combining CPI with JAK inhibition a critical message of this study and a dimension that should be more profoundly considered in derived preclinical and clinical studies to ensure the adequate therapeutic windows are applied.

While targeting members of the JAK protein family has already been reported for the treatment of multiple cancer types (16), studies addressing its consequences in the immune system have been scarce and even partially discouraging. This is probably due to the logical notion that inhibiting a pivotal immunostimulatory cytokine like IFN-γ should have detrimental effects in ongoing antitumor responses, supported by reported findings revealing acquired resistance to immunotherapy upon loss of JAK function (40–43). However, we and others have described the unwanted consequences of uncontrolled immunostimulation (3, 6, 7), possibly mediated by compensatory immunoregulatory mechanisms to prevent tissue damage, and emerging evidence highlights JAK/STAT as a pivotal pathway in sustained T cell exhaustion (19, 28). In the case of CPI, related toxicities observed in mice and patients are no more than a manifestation of such unwanted effects (44). Strategies aiming to balance out immune stimulation without compromising antitumor efficacy are therefore bound to improve not only response rates but also quality of life for patients undergoing immunotherapeutic treatments. In fact, other examples of immunotherapeutic advantage by targeting an immunostimulatory molecule (TNF-α) in preclinical models have been published (45). In the present study, we demonstrate that early targeting of JAK1 in the prostate model TRAMP-C2 by treating with ABT-317 after dual CPI induces optimal expansion of cytotoxic, antigen-specific T cells that leads to toxicity-free, tumor-free, rechallenge-proof responses in mice. Because TRAMP-C2 tumors take around 40 days after implantation and there was no analyzable tumor tissue from best responding mice in these experiments, we focused our efforts in the multiomic assessment of early immune antitumor events upon JAK inhibition on TDLNs, as well as in ex vivo susceptibility for restimulation of TDLN lymphocytes from treated mice to discern the immunomodulation exerted by ABT-317 leading to tumor rejection. Our ex vivo and in vivo findings concur on the beneficial suppression of IFN-γ signaling exerted by early and sequential ABT-317. While the most relevant advantages become obvious in a delayed manner, both at the macroscopic (tumor control, resistance to tumor rechallenge) and molecular level (delayed antigen-specific CD8^+^ T cell burst), we were also able to detect early immune-related events attributable to treatment with ABT-317, such as the decoupling of CPI-enriched IFN-γ and cell death gene signatures and their related signaling pathways in key antitumor cell populations such as effector CD8^+^ and CD4^+^ Tregs; improved ex vivo preparedness for IFN-γ release; and unaffected cell viability, proliferation, and cytolytic potential upon both indiscriminate or antigen-specific restimulation of TDLN lymphocytes. In both tumor models described in the study, we were able to capture the improved fitness provided by the combination of CPI with sequential ABT-317 in antigen-specific SPAS-1–reactive (TRAMP-C2) and ADPGK-reactive (MC38) CD8^+^ T cell responses, with increased frequencies and effector profiles and reduced exhaustion. We believe these findings regarding T cell dynamics are important additions to the advantages of combining delayed concomitant JAK inhibition and CPI described in preclinical melanoma models (7) and describe an unprecedented role for JAK inhibition in subverting immune-intrinsic resistance to immunotherapy.

Notably, we observed increased memory-like T cell populations in the TDLNs of rechallenged mice only when originally treated with combined CPI and ABT-317, thus verifying its long-lasting benefits. We validated these findings in the faster growing colorectal MC38 tumor model using multiparameter flow cytometry and scRNA-Seq in a minimal tumor burden setting, where treatment with ABT-317 significantly attenuated CPI-induced IFN-γ molecular signatures (Figure 6), prevented Treg expansion, and set the stage for a delayed boost in tumor-infiltrating antigen-specific CD8^+^ T cells with high proliferation and effector capabilities. Interestingly, self-renewal PD-1^+^TCF1^+^CD8^+^ T cells were depleted in CPI-treated animals in this model. We believe the more moderate benefit provided by ABT-317 in terms of tumor growth and survival in the MC38 model underscores the time-dependent therapeutic window proposed for ABT-317 in combination with CPI.

Our study presents a handful of limitations. First, although we consider the TRAMP-C2 ETB setting an important and underexplored field in immunotherapy and very relevant to the study of prostate cancer, the use of a different cancer model for tumor immunophenotyping partially limits the strength of our conclusions regarding prostate-specific antitumor changes. We addressed this caveat with rechallenge studies in mice that had completely rejected tumors, but we could also be missing crucial immune-relevant events happening at the very beginning of tumor formation, such as myeloid reprogramming, which could in turn be relevant in more advanced stages of malignancy, when critical masses of lymphoid and myeloid cells have been recruited into the tumor and when disease detection is likely more frequent in patients. Additionally, our toxicity assessment was limited to body weight variation, although experimental design did not include change in percentage body weight randomization prior to treatment with ABT-317. Moreover, while we describe strong immune-intrinsic consequences of combined CPI and JAK inhibition in our prostate tumor model, we cannot completely rule out additional tumor-intrinsic responses to ABT-317 taking place, such as previously described restriction of tumor cell plasticity in prostate cancer (46). Further studies using additional prostate tumor models — ideally orthotopic, still a gap in the field — with naturally or manipulated varying levels of JAK expression will be pivotal to elucidate these questions.

Two recent clinical studies have combined JAK inhibition with CPI in patients with Hodgkin lymphoma (17) and lung cancer (18), in addition to a recent preclinical study showing prevention of cytokine-induced CAR T cell exhaustion in acute myeloid leukemia (19). While the focus of our study is on T cell responses and our approach to the myeloid compartment was limited, we had parallel observations to the increase in DCs, and specifically CCR7^+^ mreg DCs, described by Zak and colleagues (17). Similarly, our work expands the findings from Mathew and colleagues (18), mainly regarding the clonal expansion of allegedly “antigen-driven” CD8^+^ T cells, evidenced by the delayed burst of more effector, less exhausted TDLN CD8^+^ SPAS-1–reactive and tumor-infiltrating ADPGK-reactive T cells in the 2 models described in this study. It is noteworthy that the JAK1/2 inhibitor ruxolitinib described in those studies appears to have strong immunomodulatory activity on its own, while ABT-317 alone did not show significant differences in this study. While this could suggest reduced immune leakiness from solely targeting JAK1, future comprehensive safety-oriented comparisons between different JAK inhibitor candidates and varying tumor type dependencies to different members of the JAK protein family should also be considered for the intelligent design of JAK-inhibiting drugs (16, 47). In summary, our observations regarding the safety and lymphoid-sparing features of ABT-317 in combination with CPI, while preclinical, are important contributions to highlighting JAK inhibition as a promising clinical companion in cancer immunotherapy and could help improve immune cell therapies that have been shown to necessitate IFN-γ to cure solid tumors, such as CAR T cells (2).

## Methods

### Sex as a biological variable.

Because the study primarily focuses on a mouse prostate tumor model, only male mice were used.

### Cell lines.

Murine prostate adenocarcinoma TRAMP-C2 (CRL-2731, ATCC) and murine colorectal adenocarcinoma MC38 (CVCL_B288, donated by Jeff Bluestone at UCSF, San Francisco, California, USA) cell lines were used in this study. They were cultured in complete Dulbecco’s modified Eagle medium (DMEM) (11995-065, Gibco) supplemented with 10% fetal bovine serum (FBS) (35-011-CV, Corning) and 1× Penicillin-Streptomycin (10,000 mL Streptomycin SO_4_, 10,000 U/mL Penicillin G). Medium supplements were obtained from the UCSF Cell Culture Facility. Both lines were certified at least yearly by the STR profiling method available at ATCC. Cells were also routinely tested for Mycoplasma contamination using the Mycoplasma PCR Detection Kit (G238, Abm).

### Animal studies.

Eight- to 10-week-old male WT C57BL/6J mice (000664, Jackson Laboratory) or *C.129S4(B6)-Ifngtm3.1Lky/J* IFN-γ–YFP reporter Great/SMART mice (donated by Richard Locksley at UCSF, San Francisco, California, USA; C57BL/6J background) were used in mouse experiments described in this study. For in vivo tumor studies, 1 million TRAMP-C2 cells were implanted subcutaneously (s.c.) on the right flank of each animal (*n* = 5) on day 0. Animals were randomized and treated intraperitoneally (i.p.) on days 3, 6, and 9 with 8 mg/kg of isotype control (IgG2a/k, AbbVie) or dual CPI anti-CTLA4 (clone 24H2, AbbVie) and anti–PD-1 (clone 17D2, AbbVie), followed by Dulbecco’s PBS (DPBS) or ABT-317 administration on days 12, 15, and 21. ABT-317 (AbbVie) is a JAK1-selective inhibitor with reported activity in preclinical models of autoimmune disease (21, 22). ABT-317 was resuspended from lyophilized powder in DPBS at 5 mg/mL. ABT-317 was administered i.p. to mice with a starting dose of 5 or 20 mg/kg on day 12 after tumor implantation, followed by halved maintenance doses on days 15 and 21. Tumors from up to *n* = 3 mice per experimental group were harvested upon reaching endpoint and processed for flow cytometry.

Tumors were measured twice a week in length (L) and width (W), where L was the higher measured value, and W was the lower. Tumor volume was obtained with the formula V = L × W^2^ × π / 6. A tumor volume of 2,000 mm^3^ or a body condition score ≤ 2 were applied as endpoints. All mice were maintained at the UCSF vivarium at all times and received food and water ad libitum. For tumor studies shown in Figure 6, mice implanted with MC38 cells were randomized into groups upon reaching 50 mm^3^, which was 7–10 days after implantation for all involved mice. Experiment performers were not blinded to experimental conditions, but measurements and treatments were performed by different operators. No data were excluded.

### Tumor rechallenge.

Surviving mice with no measurable tumors at day 80 after implantation across *n* = 2 experiments were challenged a second time with either 1 million TRAMP-C2 (*n* = 8) or 1 million MC38 cells (*n* = 6) on their left flank. Tumors were measured twice a week, and TRAMP-C2 tumors were harvested at day 85 after rechallenge (day 40 or when reaching endpoint volumes for MC38) for immunophenotyping by flow cytometry. No further treatment was administered to these mice.

### Time course immunophenotyping.

A total of 1 million TRAMP-C2 cells were injected s.c. on the right flank of mice. Animals were treated on days 3, 6, and 9 with isotype control, anti-CTLA4 (clone 24H2), anti–PD-1 (clone 17D2), and/or ABT-317 starting day 12. On days 12 (*n* = 5), 15 (*n* = 5), 21 (*n* = 5), and 25 (*n* = 5) after implantation, lymph nodes were surgically resected and processed for immunophenotyping by flow cytometry.

### Tissue processing.

Spleens, inguinal TDLNs, and tumors were surgically removed with sterilized equipment. Spleens were mechanically dissociated through a 70 μm filter (223635548, Thermo Fisher Scientific) into a 50 mL conical tube with cold PBS. Lymph nodes were mechanically dissociated between 2 super-frosted microscope slides and washed into wells of 6-well plates containing cold PBS. Tumors were minced with scalpel blades and digested to single-cell suspensions by incubation for 1 hour at 37°C in tumor digestion media containing DMEM, 10% FBS, 2 mg/mL Collagenase IV (C5138, MilliporeSigma), and DNase I (D5025, MilliporeSigma). Then, tumor lysates were filtered through a 100 μm filter (431752, Corning) into 50 mL conical tubes and filled with cold PBS. Tissue single-cell lysates were centrifuged at 450*g* for 5 minutes at 4°C. Supernatants were discarded and pellets were resuspended in 5 mL ACK Lysing Buffer (118-156-101, Quality Biological), mixed well, and kept on ice for 5 minutes. Lysis was stopped by filling the tubes with cold PBS. Samples were centrifuged again at 450*g* and finally resuspended in 1 mL cold PBS. Viable cells for downstream use were counted in a Vi-CELL cell counter (Beckman Coulter) at a 1:60 dilution.

### Immunofluorescence.

Immunostaining of mouse tissues was performed on 5 μm acetone-fixed cryosections. Briefly, sections of inguinal lymph nodes from tumor-bearing mice were immunostained with the following antibodies: anti-mouse CD11c–Alexa Fluor 647 (N418, BioLegend) at a 1:100 dilution, rabbit anti-mouse CD86 antibody (E5W6H, Cell Signaling Technology) at a 1:100 dilution, followed by donkey anti-rabbit IgG-PE (Poly4064, BioLegend) at a 1:200 dilution and rat anti-mouse DC-LAMP antibody (1006F7.05, Novus Biologicals) at a 1:100 dilution, followed by goat anti-rat IgG–Alexa Fluor 647 (Poly4054, BioLegend) at a 1:200 dilution. After staining, slides were washed, counterstained with DAPI to visualize nuclei, and mounted using ProLong Gold Antifade Mountant (P36930, Invitrogen). Images were captured using a Leica DMi8 microscope equipped with a 63×/1.32 oil objective and a Leica DFC9000 GTC digital microscope camera, operated with LAS X software (v3.5.7.23225). Image processing to generate fluorescent channel overlays and confirm uniform exposure adjustment was performed using ImageJ (NIH, v2.14.0/1.54f).

### Flow cytometry.

Single-cell suspensions were first incubated with 1:100 Zombie NIR (L34976, Life Technologies) for 10 minutes in the dark. After a washing step, samples were incubated with 1:100 H-2D(b) Mouse 244-252 STHVNHLHC SPAS-1 or H-2D(b) ASMTNMELM ADPGK PE-conjugated tetramers (NIH Tetramer Core) for 30 minutes at room temperature. After washing, surface staining including 1:50 Fc Block (70-0161-U500, Tonbo Biosciences) was performed for 30 minutes on ice. Cells were fixed using the eBioscience FoxP3 kit (00-5523-00, Life Technologies), and intracellular staining cocktail was finally added to samples for 30 minutes on ice before final wash with FACS Buffer (PBS containing 2% FBS and 1 mM EDTA). Samples were run on an LSR Fortessa X-50 (Becton Dickinson). Instrument day-to-day variability was adjusted for using BD FACSDiva CS&T Research Beads or Rainbow Calibration particles. MFI of the fluorescent beads was recorded for each channel on the first day of acquisition of the first samples. On following acquisition days, laser voltages were adjusted in order to match the MFI intensity acquired on the first day. After voltage adjustment, instrument compensation was recorded each day before samples acquisition using single staining UltraComp eBeads (Life Technologies). Data were analyzed by FlowJo 10.7 (TreeStar). A detailed list of antibodies used can be found in Supplemental Table 1. All antibodies used for flow cytometry were validated with relevant isotypes and fluorescence minus one. Unstained samples were also included in every run as controls.

### In vitro ABT-317 dose curve.

A total of 10^5^ naive splenocytes from C57BL/6J mice were plated onto a 96-well F-bottom plate, then stimulated with Cell Stimulation Cocktail (00-4970-93, Life Technologies) — containing PMA and ionomycin — in the presence of ABT-317 at 0, 0.125, 0.25, or 0.5 μg/μL for 4 hours at 37°C. Then, well supernatants were collected and tested for mouse IFN-γ by ELISA (MIF00, R&D Systems). Cell pellets were washed and stained for Live/Dead, CD4, CD8, and CD69, then run by flow cytometry.

### Ex vivo stimulation of lymph node cells from tumor-challenged mice.

A total of 10^5^ whole lymph node cells collected from mice on day 25 after tumor implantation were plated onto 96-well plates and stimulated with Mouse Activator CD3/CD28 Dynabeads (11456D, Life Technologies) at a 1:1 bead/cell ratio. After 48 hours, cells were washed in FACS Buffer and run by flow cytometry.

Alternatively, 10^5^ cells from day 15 lymph nodes were plated onto 96-well plates and stimulated with Cell Stimulation Cocktail (00-4970-93, Life Technologies) in the presence or absence of 0.125 μg/μL ABT-317 for 4 hours at 37°C. Then, cells were washed and stained for apoptotic annexin V using the FITC Annexin V Apoptosis Detection Kit I (556547, BD Pharmingen) and run by flow cytometry.

### Assessment of T cell death in lymphoid tissues from tumor-challenged mice.

Lymph nodes from tumor-implanted mice were harvested and processed on day 15 after implantation. Single-cell suspensions were stained for late apoptosis with 7-AAD (A1310, Thermo Fisher Scientific) and early apoptosis with annexin V using the FITC Annexin V Apoptosis Detection Kit I and run by flow cytometry.

### Ex vivo killing assays.

TDLNs from tumor-rechallenged mice were harvested and processed to single-cell suspension as described previously. Total T cells were enriched using the mouse Pan T Cell Isolation Kit II (130-095-130, Miltenyi Biotec). A total of 10^5^ T cells per well were plated in U-bottom, 96-well plates and were incubated with 5 μg/mL of SPAS-1 peptide STHVNHLHC (custom, A&ALabs) for 4 hours at 37°C in RPMI medium. Afterward, T cells were washed and were added to CytoRed-labeled target TRAMP-C2 cells at a 20:1 effector/target ratio in F-bottom, 96-well plates in the presence of Mouse Activator CD3/CD28 Dynabeads at a 1:1 bead/T cell ratio. Then, anti–Annexin V–CytoGreen was added to the wells, and target cell death was monitored every 4 hours by time-lapse microscopy (Incucyte, Sartorius). CytoRed^+^CytoGreen^+^ double-positive cells were quantified as apoptotic target cells for each condition, and values from unstimulated wells were subtracted from stimulated wells for normalization.

### scRNA-Seq.

Eight- to 10-week-old male WT C57BL/6J mice were implanted s.c. with 1 million TRAMP-C2 or MC38 cells and treated i.p. with isotype or CPI (anti-CTLA4 + anti–PD-1) on days 3, 6, and 9, followed by vehicle (DPBS) or 20 mg/kg ABT-317 on day 12. On day 15 after implantation, tumors and TDLNs from mice were harvested and processed to single-cell suspension as described in *Tissue processing*. To enrich for CD45^+^ cells in tumors, total tumor lysates were stained for Live/Dead and CD45. Live CD45^–^ or CD45^+^ (tumors) were sorted with a FACSAria (BD) into separate tubes and barcoded by with mouse TotalSeq-C03 hashtag antibodies 1–10 (155861, 155863, 155865, 155867, 155869, 155871, 155873, 155875, 155877, 155879, BioLegend) at a 1:200 dilution for 40 minutes at 4°C to identify individual samples and treatment groups. Then, individual reactions of 6 × 10^4^ cells each with 1:1 CD45^+^/CD45^–^ (tumors) ratios (unsorted total cells for TDLNs) and equal hashtag representation for batch correction purposes were prepared at the Chromium Controller (10x Genomics) using the Chromium Next GEM Single Cell 5’ Kit v2 (1000263, 10x Genomics) and the Chromium Single Cell Mouse TCR Amplification kit (1000254, 10x Genomics), and sequencing was performed on an Illumina NovaSeq 6000 with paired-end 100–base pair read lengths.

### Gene expression analysis.

Alignment and assembly of raw scRNA-Seq data were done with CellRanger versions 5 and 7 (10x Genomics, Genome Build: GRCm38 7.0.0). For further preprocessing and analysis, we used the Scanpy76 single-cell analysis toolkit (scanpy 1.8.2 and 1.9.0, pandas 1.2.4, numpy 1.20.2) in Python 3.8.12. Cells with fewer than 200 genes expressed and genes detected in less than 3 cells were excluded. For further quality control, we only used cells with <2,500 genes and <10% mitochondrial genes. Reads were normalized to 10,000 per cell, and a log + 1 transformation was applied. For visualization only, genes were subset to highly variable genes using the default Scanpy preprocessing function. The dimensionality reduction (for 2D visualization) was calculated using UMAP algorithm. Clustering was performed using the Leiden algorithm (resolution = 0.5, leidenalg 0.8.8). Gene set scores were calculated using Scanpy’s scanpy.tl.score_genes function. DGE analysis was performed with DESeq2 included in Bioconductor 3.19 (R package) (48). DEGs were cut off by adjusted *P* < 0.05 and used as input for pathway enrichment analyses against publicly available gene sets (49) performed with GSEA 4.3.2 (50).

### ABT-317 dose schedule comparison.

Eight- to 10-week-old male WT C57BL/6J mice were used in these studies. Tumor implantation was performed as described above. Mice were treated with antibodies on days 3, 6, 9. They were enrolled into the previously detailed sequential ABT-317 regime on days 12, 15, 21; a concomitant regime on days 3, 6, 12; or isotype/CPI alone. A starting ABT-317 dose of 20 mg/kg was used, followed by halved maintenance doses. In each experiment, some mice (*n* = 3) were sacrificed on day 22 after implantation and immunophenotyping was performed on TDLNs by flow cytometry as described above. The remaining mice (*n* = 7) were monitored twice/week for tumor growth and survival.

### Data analysis and statistics.

All statistical analyses were performed with GraphPad Prism 9 and 10. All data were tested for normality. For comparisons between 2 groups, Mann-Whitney statistical tests were applied. For single-parameter comparisons involving more than 2 groups, 1-way ANOVA tests adjusted for Tukey’s multiple comparisons were applied in data passing normality, while Kruskal-Wallis tests adjusted for Dunn’s multiple comparisons were applied for nonparametric data. For Kaplan-Meier survival curve comparisons, log-rank Mantel-Cox tests were applied. For scRNA-Seq, built-in Scanpy 8 statistical tools were used. A *P* value of 0.05 or less was considered significant. Unless stated otherwise, error bars in plots represent SD, and vertical/horizontal black bars represent means. Antitumor efficacy experiments were performed in triplicate (*n* = 2 for time course and scRNA-Seq). A minimum of 3 biological replicates were run in all experiments.

### Study approval.

All experiments involving animals performed in this study were reviewed and approved by the IACUC at UCSF in protocol number AN202446.

### Data availability.

All source data shown in this article and related supplemental files can be found in the Supporting Data Values spreadsheet associated with this publication. The sequencing datasets shown in this study have been deposited to NCBI GEO with accession number GSE289532.

## Author contributions

MAB, AG, WR, and LF conceived this study. WR, AG, JVG, and FAH provided critical reagents. MAB, PC, YML, A Srinath, A Lyu, RMW, DNL, MS, MC, A Lea, and A Setayesh performed experiments. ZF, MAB, and A Lyu performed bioinformatic analysis. MAB analyzed all data. MAB and LF wrote the manuscript. AG, WR, JVG, SSK, and LF provided scientific feedback, contributed to manuscript writing, and supervised the study.

## Supplementary Material

Supplemental data

Supporting data values

## Version 1 02/27/2025

In-Press Preview

## Version 2 04/08/2025

Electronic publication
