# Supplementary material for: Sequential JAK inhibition enhances antitumor immunity after combined anti–PD-1 and anti-CTLA4
Source: JCI Insight. 2025 Feb 27;10(7):e187921. doi: 10.1172/jci.insight.187921 (PMC11981626; doi:10.1172/jci.insight.187921)
Supplement: Supplemental data [file jciinsight-10-187921-s083.pdf]

Table S1. Antibodies used for flow cytometry.

Marker	Conjugation	Vendor	Catalog #	Clone
CD45	BUV661	BD	612975	30-F11
CD8α	BUV805	BD	612898	53-6.7
CD4	BV711	Biolegend	100447	GK1.5
CD3	BUV395	BD	563565	145-2C11
PD-1	PE-Dazzle594	Biolegend	109115	RMP1-30
SPAS-1 H-2Db 244-252 STHVNHLHC	PE	NIH Tetramer Core	Order#36028	N/A
ADPGK H-2Db ASMTNMELM	PE	MBL	TB-5113-1	N/A
PRF1	APC	Biolegend	154303	S16009A
Ki-67	BV480	BD	566109	B56
FoxP3	PE-Cy7	Life Technologies	25-5773-80	FJK-16s
TCF-1	A488	BD	567018	S33-966
TIM3	PerCP-Cy5.5	Biolegend	134012	B8.2C12
LAG-3	BV785	Biolegend	125219	C9B7W
CD44	BV650	Biolegend	103049	IM7
CD62L	BV570	Biolegend	104433	MEL-14
CD39	BV421	BD	567105	Y23-1185
CD69	PerCP-Cy5.5	Biolegend	104521	H1.2F3

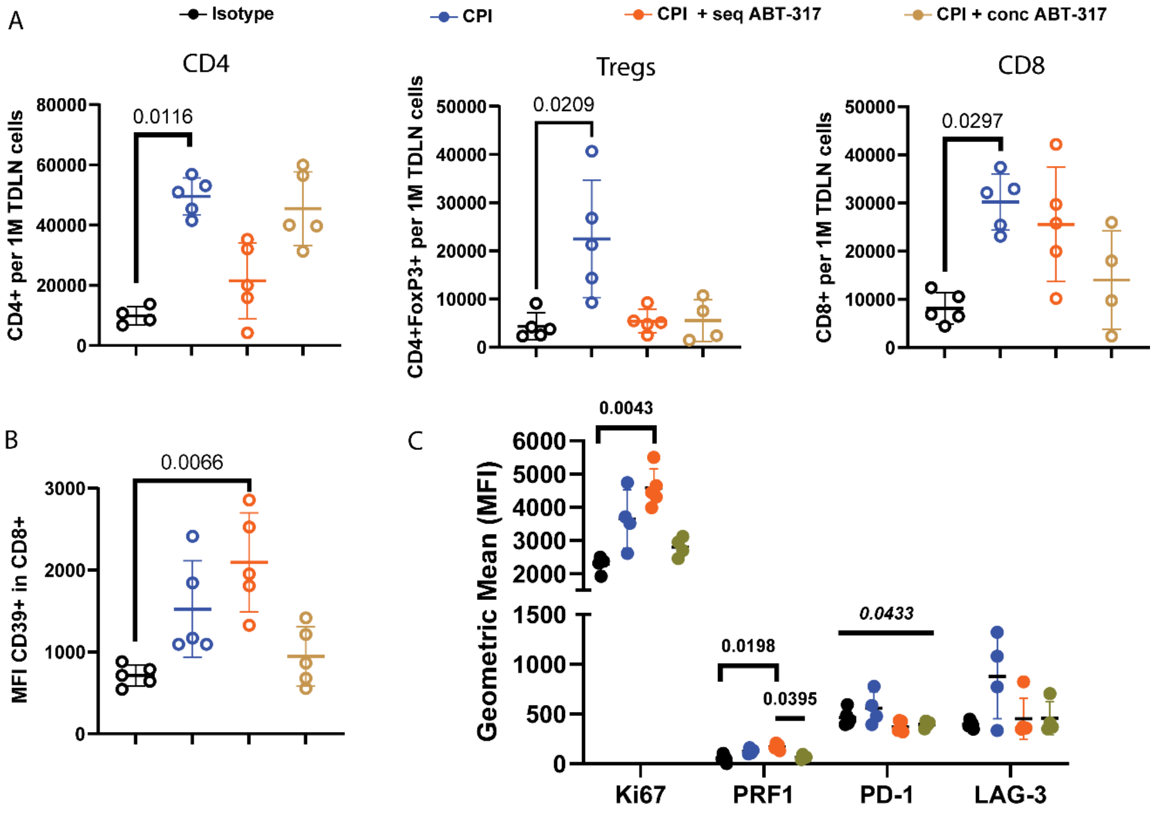

Figure S1. Treatment with ABT-317 following CPI leads to improved antitumor T cell fitness.

(A) CD4+ (left), CD4+FoxP3+ regulatory T cells (middle) and CD8+ (right) T cell counts per 1M TDLN cells obtained by flow cytometry. Significant p values by Kruskal-Wallis are shown. Error bars represent SD.

(B) CD39 MFI in TDLN CD8+ T cells. Significant p values by Kruskal-Wallis are shown. Error bars represent SD.

(C) Flow cytometry geometric mean fluorescence intensity (MFI) for Ki67, PRF1, PD-1 and LAG-3 in TDLN-derived TRAMP-C2 antigen-reactive CD8+ SPAS-1+ T cells. Significant p values by Kruskal-Wallis are shown. For PD-1, an overall p=0.0433 was calculated with no significant differences between groups. Error bars represent SD.

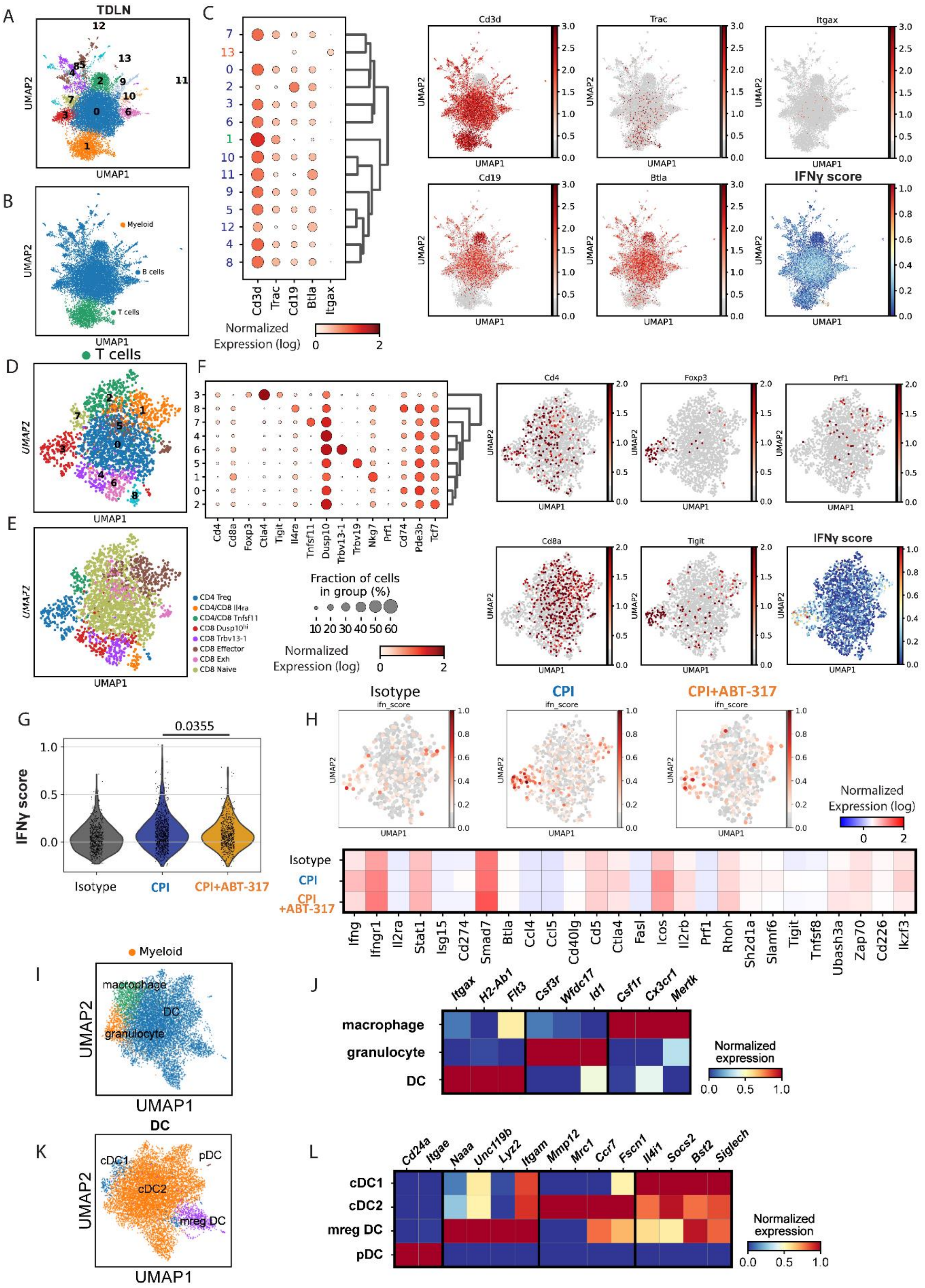
A
TDLN
UMAP1
UMAP2
B
Myeloid
B cells
T cells
C
Cd3d
Trac
Cd19
Btla
Itgax
Normalized
Expression (log)
IFNγ score
T cells
D
E
CD4 Treg
CD4/CD8 Il4ra
CD4/CD8 Tnfsf11
CD8 Dusp10hi
CD8 Trbv13-1
CD8 Effector
CD8 Exh
CD8 Naive
F
Cd4
Cd8a
Foxp3
Ctla4
Tigit
Il4ra
Tnfsf11
Dusp10
Trbv13-1
Trbv19
Nkg7
Prf1
Cd74
Pde3b
Tcf7
Fraction of cells
in group (%)
10 20 30 40 50 60
G
0.0355
IFNγ score
Isotype
CPI
CPI+ABT-317
H
ifn_score
Ifng
Ifngr1
Il2ra
Stat1
Isg15
Cd274
Smad7
Btla
Ccl4
Ccl5
Cd40lg
Cd5
Ctla4
Fasl
Icos
Il2rb
Prf1
Rhoh
Sh2d1a
Slamf6
Tigit
Tnfsf8
Ubash3a
Zap70
Cd226
Ikzf3
I
Myeloid
macrophage
DC
granulocyte
J
Itgax
H2-Ab1
Flt3
Csf3r
Wfdc17
Id1
Csf1r
Cx3cr1
Mertk
Normalized
expression
K
DC
cDC1
pDC
cDC2
mreg DC
L
Cd24a
Itgae
Naaa
Unc119b
Lyz2
Itgam
Mmp12
Mrc1
Ccr7
Fscn1
Il4i1
Socs2
Bst2
Siglech

Figure S2. ETB immune landscape of TDLNs from TRAMP-C2 challenged mice treated with ABT-317.

(A) UMAP showing 15,724 total TDLN cells from at least n=3 mice per treatment group for scRNAseq analysis from n=2 experiments described in Fig. 2F. 14 phenotypic clusters were obtained by unsupervised leiden clustering (res: 0.5).

(B) UMAP showing major B cell, Myeloid and T cell populations.

(C) Dot plot showing z-scaled, normalized expression of lineage B cell, Myeloid and T cell markers by leiden phenotypic cluster. UMAPs showing normalized expression of lineage-defining *Cd3d, Trac, Itgax, Cd19, Btla* and IFNγ score.

(D) UMAP showing 2,153 TDLN T cells from cluster 1 in (A). 9 phenotypic clusters were obtained by unsupervised leiden clustering (res: 0.5).

(E) UMAP showing phenotypic clusters by CD4 / CD8 expression and top-rank gene expression.

(F) Dot plot showing z-scaled, normalized expression of cluster-defining TDLN T cell genes by leiden phenotypic clustering. UMAPs showing normalized expression of selected markers *Cd4, Cd8a, Foxp3, Prf1, Tigit* and IFNγ score.

(G) Violin plots showing per-cell IFNγ score in TDLN T cells. Significant p value obtained by Tukey is shown.

(H) UMAPs showing the normalized IFNγ score ('ifn_score') for TDLN T cells interrogated with a 26-gene IFNγ-responsive gene signature in each experimental group. Heatmap showing normalized expression of each of the 26 genes scored in 'IFNγ score' in all TDLN T cells, by treatment.

(I) UMAP showing TDLN myeloid cells by major cell type.

(J) Heatmap showing normalized expression of macrophage, granulocyte and dendritic cell (DC) lineage markers.

(K) UMAP showing 8,412 TDLN DCs from experiments described in Fig. 2F populating 4 different phenotypic clusters by leiden (res. 0.5). cDC: Conventional Dendritic Cells; pDC: Plasmacytoid Dendritic Cells; mregDC: Mature immunoregulatory Dendritic Cells.

(L) Heatmap showing normalized expression of TDLN DC subset lineage markers.

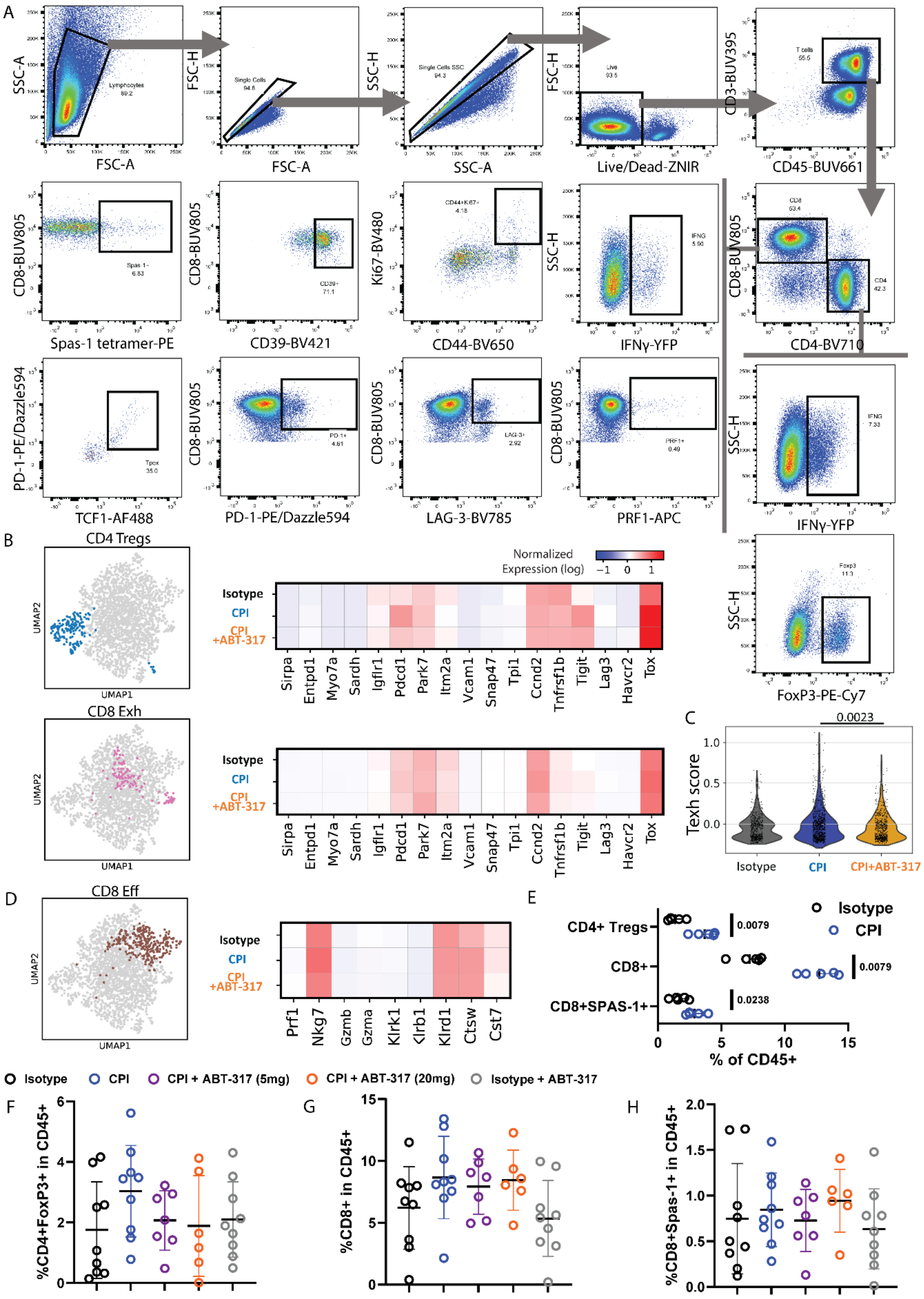

A
SSC-A
FSC-A
Lymphocytes 89.2
FSC-H
Single Cells 94.8
SSC-H
Single Cells SSC 94.3
SSC-A
Live 93.5
Live/Dead-ZNIR
CD3-BUV395
T cells 55.5
CD45-BUV661
CD8-BUV805
Spas-1 tetramer-PE
CD39-BV421
Ki67-BV480
CD44-BV650
IFNγ-YFP
CD4-BV710
PD-1-PE/Dazzle594
TCF1-AF488
Tpex 35.0
PD-1-PE/Dazzle594
LAG-3-BV785
PRF1-APC
SSC-H
IFNγ-YFP
FoxP3-PE-Cy7
B
CD4 Tregs
CD8 Exh
Normalized Expression (log)
Isotype
CPI
CPI +ABT-317
Sirpa Entpd1 Myo7a Sardh Igflr1 Pdcd1 Park7 Itm2a Vcam1 Snap47 Tpi1 Ccnd2 Tnfrsf1b Tigit Lag3 Havcr2 Tox
UMAP1
UMAP2
C
0.0023
Texh score
Isotype
CPI
CPI+ABT-317
D
CD8 Eff
Prf1 Nkg7 Gzmb Gzma Klrk1 Klrb1 Klrd1 Ctsw Cst7
E
CD4+ Tregs
CD8+
CD8+SPAS-1+
0.0079
0.0079
0.0238
% of CD45+
Isotype
CPI
CPI + ABT-317 (5mg)
CPI + ABT-317 (20mg)
Isotype + ABT-317
F
%CD4+FoxP3+ in CD45+
G
%CD8+ in CD45+
H
%CD8+Spas-1+ in CD45+

Figure S3. Harnessing antitumor T cell fitness in ETB TRAMP-C2.

(A) Flow cytometry gating strategy for all experiments described in the study. Relevant isotypes and irrelevant tetramers were used for titration. Values represent percentages of parent.

(B) Left, UMAPs showing TDLN CD4+ Tregs (cl. 3) (top) and CD8+ Exhausted (cl. 5) T cells. Right, heatmaps showing normalized expression of 17 genes associated with T cell exhaustion composing the 'Texh score'.

(C) 'Texh scores' in day 15 TDLN T cells. Significant p value obtained by Tukey is shown.

(D) Left, UMAP showing TDLN CD8+ Effector T cells (cl. 1). Right, heatmap showing normalized expression of effector T cell genes.

(E) Percentage of CD4+ Tregs, overall CD8+ and CD8+SPAS-1+ T cells within immune CD45+ cells by flow cytometry from TDLNs harvested on day 12 post TRAMP-C2 implantation on mice treated intraperitoneally with either dual CPI or Isotype on days 3, 6 and 9. Significant P values by Mann-Whitney are shown. Small black bars represent Mean. Error bars represent SD.

(F) Flow cytometry tumor endpoint data from ETB experiments described in Fig. 2A showing percentage of tumor-infiltrating Tregs within CD45+ cells. No significant differences were found by Kruskal-Wallis and Dunn tests. Horizontal bars represent mean. Error bars represent SD.

(G) Same as (F), for overall CD8+ T cells.

(H) Same as (F), for antigen-reactive CD8+SPAS-1+ T cells.

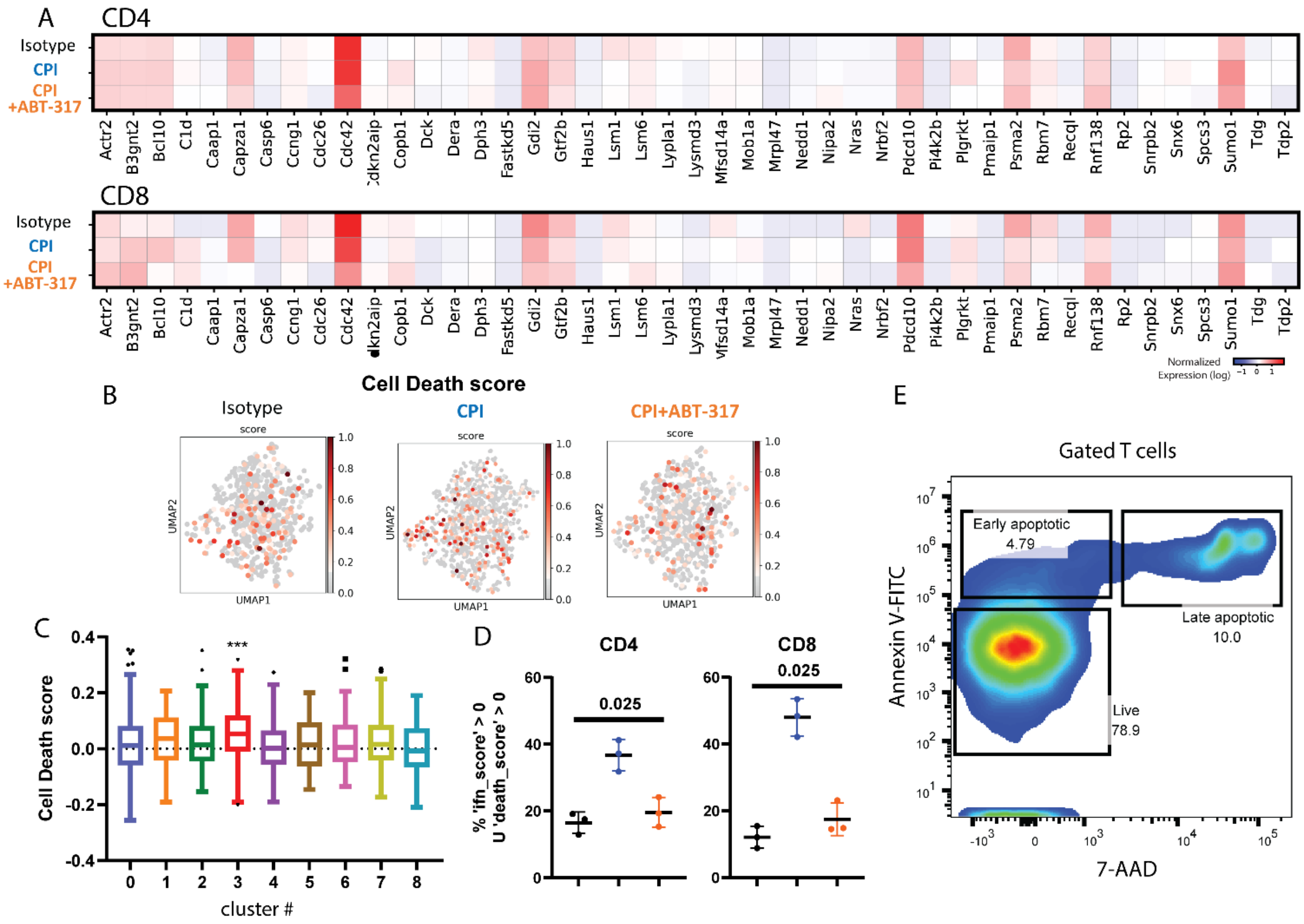

Figure S4. ABT-317 prevents AICD in CD4+ and CD8+ T cells.

(A) Heatmap showing normalized expression of 45 genes composing the 'Cell death score' in TDLN from mice harvested on day 15 after tumor implantation, by CD4 or CD8 lineage and treatment group.

(B) UMAPs showing 'Cell death score' in day 15 TDLN T cells, by treatment.

(C) Cell death score by leiden phenotypic cluster in TDLN T cells. ***, $p < 0.05$ vs clusters 0, 2, 4, 7, 8 by Tukey. Boxes cover interquartile interval, split by the median. Whiskers represent Min to Max values. Tukey outliers are shown.

(D) T cells contained in gates (in purple) shown in Fig. 4B from each independent scRNAseq sample, split by CD4 or CD8 lineage. Significant P values obtained by Kruskal-Wallis are shown. Black horizontal bars represent mean. Error bars represent SD. Black: Isotype; Blue: CPI; Orange: CPI+ABT-317.

(E) Flow cytometry gating for assessment of T cell death in day 15 TDLNs (Fig. 4D-E) and ex vivo experiments shown in Fig. 4E. Values represent percentages of parent.

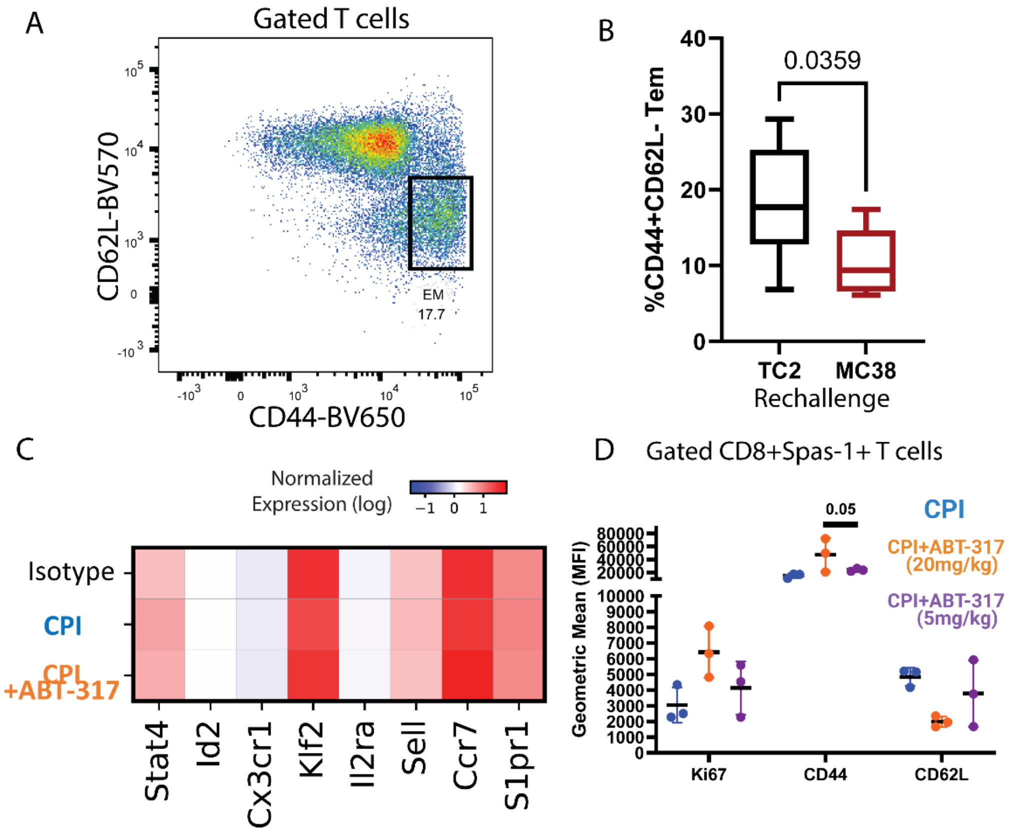

Figure S5. ABT-317 protects mice from immunological rechallenge.

(A) Flow cytometry gating for CD44 (x axis) and CD62L (y axis) in TDLN-derived T cells from rechallenged mice involved in Fig. 5A. Values represent percentages of parent.

(B) Box plot showing percentage of CD44+CD62L- Tem cells in TDLNs from rechallenged mice, split by tumor cell line used for rechallenge. Significant P value by Mann-Whitney is shown. Boxes span 10-90 percentiles. Horizontal bar across boxes represents median. Error bars represent SD.

(C) Heatmap showing normalized expression of Effector Memory T cell marker genes in day 15 TDLNs from mice involved in experiments described in Fig. 2F, by treatment.

(D) Geometric mean fluorescence intensity (MFI) of Ki67, CD44 and CD62L in CD8+ SPAS-1+ T cells from TDLNs of TRAMP-C2-rechallenged mice, by original treatment group (Fig. 2A).

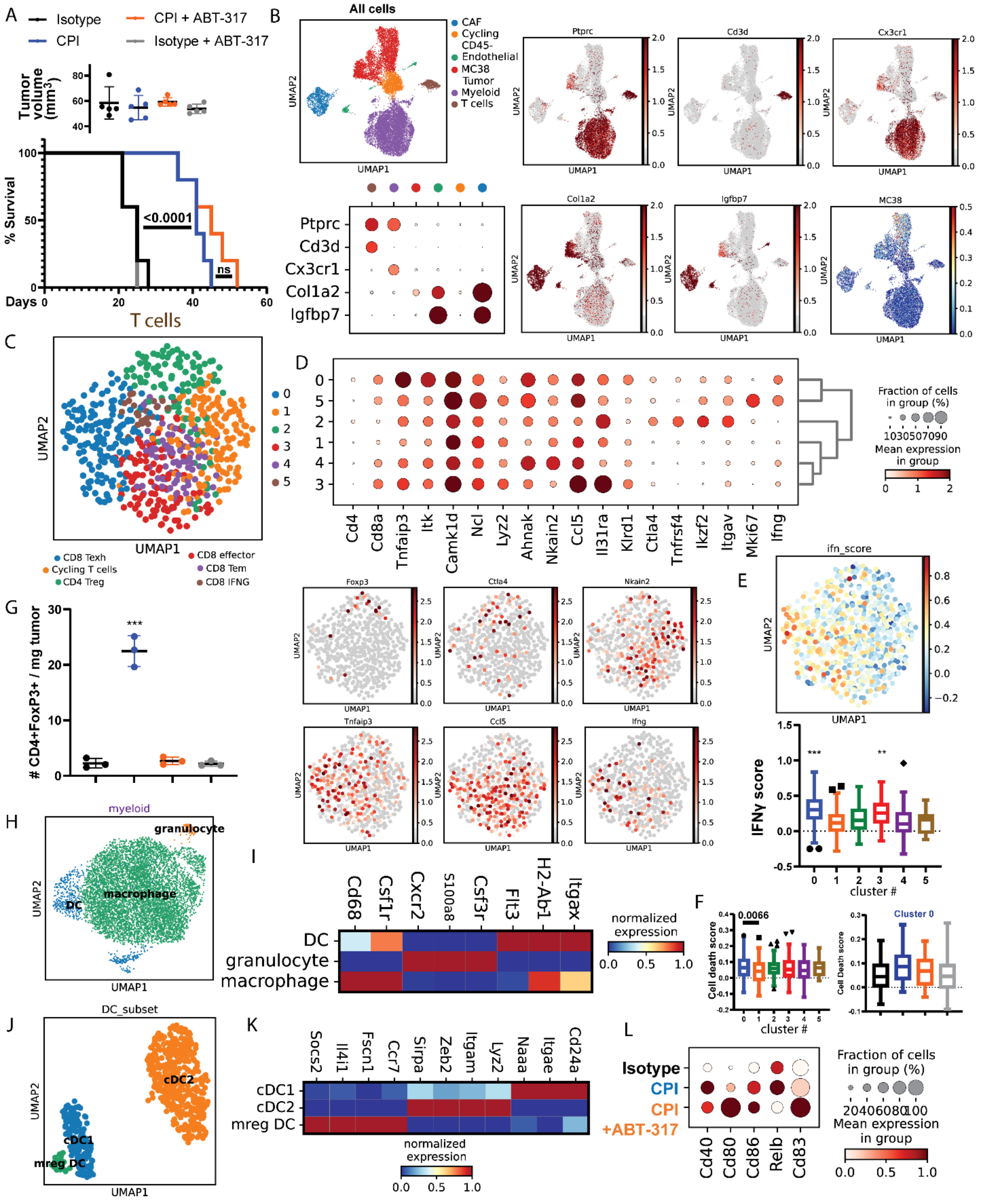

A
Isotype
CPI
CPI + ABT-317
Isotype + ABT-317
Tumor volume (mm3)
% Survival
<0.0001
ns
Days
B
All cells
CAF
Cycling CD45-
Endothelial
MC38 Tumor
Myeloid
T cells
UMAP1
UMAP2
Ptprc
Cd3d
Cx3cr1
Col1a2
Igfbp7
MC38
T cells
C
CD8 Texh
Cycling T cells
CD4 Treg
CD8 effector
CD8 Tem
CD8 IFNG
D
Cd4
Cd8a
Tnfaip3
Itk
Camk1d
Ncl
Lyz2
Ahnak
Nkain2
Ccl5
Il31ra
Klrd1
Ctla4
Tnfrsf4
Ikzf2
Itgav
Mki67
Ifng
Fraction of cells in group (%)
Mean expression in group
Foxp3
Ctla4
Nkain2
Tnfaip3
Ccl5
Ifng
E
ifn_score
IFNγ score
cluster #
G
CD4+FoxP3+ / mg tumor
***
F
Cell death score
0.0066
Cluster 0
H
myeloid
granulocyte
macrophage
DC
I
Cd68
Csf1r
Cxcr2
S100a8
Csf3r
Flt3
H2-Ab1
Itgax
normalized expression
J
DC_subset
cDC1
cDC2
mreg DC
K
Socs2
Il4i1
Fscn1
Ccr7
Sirpa
Zeb2
Itgam
Lyz2
Naaa
Itgae
Cd24a
L
Isotype
CPI
CPI +ABT-317
Cd40
Cd80
Cd86
Relb
Cd83

Figure S6. ABT-317 improves antitumor T cell fitness in a minimal tumor burden setting.

(A) Top, Treatment randomization tumor volumes (day 0) for mice involved in experiments described in Fig. 6A. Black horizontal bar represents median. Error bars represent SD. Bottom, survival analysis for MC38 experiments described in Fig. 6A. Statistical significance by Log-rank Mantel-Cox analysis is shown: $p < 0.0001$ CPI and CPI+ABT317 vs. Isotype and Isotype vs ABT-317. NS= not significant CPI vs CPI+ABT-317 ($p = 0.187$).

(B) UMAP showing 30,312 total cells for scRNAseq analysis from MC38 tumors harvested as described in Fig. 6A, by major cell types. At least n=3 mice per treatment group are represented. Below, dot plot matrix showing z-scaled, normalized expression of top-rank genes by leiden phenotypic major cell clusters. Right, UMAPs showing normalized gene expression levels of immune cell *Ptprc* (CD45), lymphoid *Cd3d* (CD3), myeloid *Cx3cr1*, fibroblastic *Col1a2*, endothelial *Igfbp7*, and a scored 'MC38 gene signature'.

(C) UMAP showing 2,432 MC38 tumor-infiltrating T cells. 6 phenotypic clusters were obtained by leiden (res: 0.5).

(D) Top, dot plot showing z-scaled, normalized expression of top-rank genes by leiden phenotypic T clusters shown in (C). Bottom, UMAPs showing normalized expression of T cell cluster-defining *Foxp3, Ctla4, Nkain2, Tnfaip3, Ccl5* and *Ifng*.

(E) Top, UMAP showing IFNγ score in tumor-infiltrating T cells. Bottom, IFNγ scores per intratumoral T cell phenotypic cluster. ***, $p \leq 0.0013$ vs clusters 1, 2, 4, 5 by Tukey; **, $p \leq 0.0001$ vs clusters 1, 4 by Tukey. Tukey outliers are shown.

(F) Left, Cell death scores per intratumoral T cell phenotypic cluster. Significant p value by Tukey is shown. Tukey outliers are shown. Right, Cell death scores in CD8+ T cell cluster 0, split by treatment. No significance by Tukey ($p = 0.065$). Boxes cover interquartile interval, split by the median. Whiskers represent Min to Max values.

(G) Counts per mg of tumor of CD4+FoxP3+ Tregs in tumors from mice involved in flow cytometry experiments described in Fig. 6A. Black horizontal bar represents mean. Error bars represent SD.

(H) UMAP showing 7,928 tumor-infiltrating myeloid cells, by major cell types.

(I) Heatmap showing normalized expression of macrophage, granulocyte and DC markers in MC38 tumor myeloids.

(J) UMAP showing 538 tumor-infiltrating DCs, by DC subsets.

(K) Heatmap showing normalized expression of tumor-infiltrating DC subset markers.

(L) Dotplot matrix showing normalized gene expression of *Cd40, Cd80, Cd86, Relb* and *Cd83* in tumoral mregDCs.
